# Association of Lipidome Remodeling in the Adipocyte Membrane with Acquired Obesity in Humans

**DOI:** 10.1371/journal.pbio.1000623

**Published:** 2011-06-07

**Authors:** Kirsi H. Pietiläinen, Tomasz Róg, Tuulikki Seppänen-Laakso, Sam Virtue, Peddinti Gopalacharyulu, Jing Tang, Sergio Rodriguez-Cuenca, Arkadiusz Maciejewski, Jussi Naukkarinen, Anna-Liisa Ruskeepää, Perttu S. Niemelä, Laxman Yetukuri, Chong Yew Tan, Vidya Velagapudi, Sandra Castillo, Heli Nygren, Tuulia Hyötyläinen, Aila Rissanen, Jaakko Kaprio, Hannele Yki-Järvinen, Ilpo Vattulainen, Antonio Vidal-Puig, Matej Orešič

**Affiliations:** 1 Department of Medicine, Division of Internal Medicine, and Department of Psychiatry, Obesity Research Unit, Helsinki University Central Hospital, Helsinki, Finland; 2 Department of Public Health, Hjelt Institute, University of Helsinki, Helsinki, Finland; 3 Institute for Molecular Medicine Finland, Helsinki, Finland; 4 Department of Physics, Tampere University of Technology, Tampere, Finland; 5 VTT Technical Research Centre of Finland, Espoo, Finland; 6 Institute of Metabolic Science, Metabolic Research Laboratories, University of Cambridge, Addenbrooke's Hospital, Cambridge, United Kingdom; 7 Department of Computational Biophysics and Bioinformatics, Jagiellonian University, Kraków, Poland; 8 Department of Medical Genetics, University of Helsinki, Helsinki, Finland; 9 Department of Mental Health and Substance Abuse Services, National Institute for Health and Welfare, Helsinki, Finland; 10 Division of Diabetes, Department of Medicine, Helsinki University Central Hospital, Helsinki, Finland; 11 Department of Applied Physics, School of Science and Technology, Aalto University, Espoo, Finland; 12 MEMPHYS—Center for Biomembrane Physics, University of Southern Denmark, Odense, Denmark; University of Calgary, Canada

## Abstract

Identification of early mechanisms that may lead from obesity towards complications such as metabolic syndrome is of great interest. Here we performed lipidomic analyses of adipose tissue in twin pairs discordant for obesity but still metabolically compensated. In parallel we studied more evolved states of obesity by investigating a separated set of individuals considered to be morbidly obese. Despite lower dietary polyunsaturated fatty acid intake, the obese twin individuals had increased proportions of palmitoleic and arachidonic acids in their adipose tissue, including increased levels of ethanolamine plasmalogens containing arachidonic acid. Information gathered from these experimental groups was used for molecular dynamics simulations of lipid bilayers combined with dependency network analysis of combined clinical, lipidomics, and gene expression data. The simulations suggested that the observed lipid remodeling maintains the biophysical properties of lipid membranes, at the price, however, of increasing their vulnerability to inflammation. Conversely, in morbidly obese subjects, the proportion of plasmalogens containing arachidonic acid in the adipose tissue was markedly decreased. We also show by in vitro Elovl6 knockdown that the lipid network regulating the observed remodeling may be amenable to genetic modulation. Together, our novel approach suggests a physiological mechanism by which adaptation of adipocyte membranes to adipose tissue expansion associates with positive energy balance, potentially leading to higher vulnerability to inflammation in acquired obesity. Further studies will be needed to determine the cause of this effect.

## Abstract 2

The authors describe a new approach to studying cellular lipid profiles and propose a compensatory mechanism that may help maintain the normal membrane function of adipocytes in the context of obesity.

## Author Summary

Obesity is characterized by excess body fat, which is predominantly stored in the adipose tissue. When adipose tissue expands too much it stops storing lipid appropriately. The excess lipid accumulates in organs such as muscle, liver, and pancreas, causing metabolic disease. In this study, we aim to identify factors that cause adipose tissue to malfunction when it reaches its limit of expansion. We performed lipidomic analyses of human adipose tissue in twin pairs discordant for obesity—that is, one of the twins was lean and one was obese—but still metabolically healthy. We identified multiple changes in membrane phospholipids. Using computer modeling, we show that “lean” and “obese” membrane lipid compositions have the same physical properties despite their different compositions. We hypothesize that this represents allostasis—changes in lipid membrane composition in obesity occur to protect the physical properties of the membranes. However, protective changes cannot occur without a cost, and accordingly we demonstrate that switching to the “obese” lipid composition is associated with higher levels of adipose tissue inflammation. In a separate group of metabolically unhealthy obese individuals we investigated how the processes that regulate the “lean” and “obese” lipid profiles are changed. To determine how these lipid membrane changes are regulated we constructed an *in silico* network model that identified key control points and potential molecular players. We validated this network by performing genetic manipulations in cell models. Therapeutic targeting of this network may open new opportunities for the prevention or treatment of obesity-related metabolic complications.

## Introduction

Obesity is characterized by excess body fat, which is predominantly stored in the adipose tissue. Obesity is considered one of the pathological features of metabolic syndrome (MetS), which also includes insulin resistance, hypertension, and dyslipidemia [1]. Although not all obese individuals develop metabolic and cardiovascular complications, the clustering of these conditions of MetS suggests there may be pathogenic mechanisms common to all these phenotypes [2],[3].

The specific mechanisms that may lead from obesity towards the higher risk of metabolic complications such as insulin resistance and type 2 diabetes (T2D) remain elusive. Our preferred nonexclusive hypothesis is the “adipose tissue expandability” hypothesis [2],[4], which states that obesity-associated metabolic complications such as insulin resistance are due to the finite capacity of adipose tissue to expand and therefore to store energy. In fact, once this limit of expansion is reached and the storage capacity of adipose tissue is exceeded, the lipids become deposited ectopically, leading to potentially toxic effects in peripheral tissues via the excessive accumulation of reactive lipid species.

In support of the pathogenic role of limited adipose tissue expandability and functionality we have previously shown in a genetically obese leptin-deficient mouse (ob/ob) model that ablation of adipogenic PPARγ2 (the POKO mouse) leads to a 65% decrease in adipose tissue mass as compared to ob/ob mouse, accumulation of reactive lipids in pancreatic islets, liver, and muscle, and severe insulin resistance and diabetes [5]. Interestingly, adipose tissue of the POKO mouse is also characterized by an altered membrane phospholipid profile, characterized by diminished levels of plasmalogens, the most abundant ether lipids [5]. The opposite experimental paradigm, increasing the capacity of adipose tissue expansion by overexpressing adiponectin in white adipose tissue of ob/ob mice (the AdTG-ob/ob mouse), led to a considerably improved metabolic profile as compared to ob/ob mouse [6]. Based on this evidence we hypothesized that adaption to the demands posed by positive-energy-balance-induced adipose tissue expansion may challenge the homeostatic mechanisms controlling phospholipid composition and their associated biophysical properties. However, given the importance of membrane lipid composition and fluidity to maintain the topology, mobility, or activity of membrane-bound proteins, and to ensure normal cellular physiology, we also speculated that the process of adipose tissue expansion should also incorporate allostatic adaptations aiming to maintain membrane functionality for as long as possible [7], particularly under metabolically challenging conditions. In this respect we speculated that exhaustion of these allostatic adaptations may determine the maximal limit of expansion and may coincide with metabolic perturbations.

Investigations of adipose tissue “lipidome,” covering a global profile of structurally and functionally diverse lipids, provide a unique opportunity to pursue accurately and sensitively studies profiling hundreds of molecular lipids in parallel [8]. The spatial complexity of lipid metabolism presents a challenge for the study of the global lipidomic profiles. Studies of lipidomic profiles in the context of biochemical pathways may shed light on processes behind the synthesis of or regulation by specific lipids, but cannot address how these changes translate into lipid membrane properties and affect cellular physiology. However, molecular modeling tools and large scale computing capacities are becoming available that facilitate modeling of lipid bilayers in the context of their composition and function [9], thus opening new opportunities to interpret regulation and changes of global lipidome also in the spatial and physiological context.

Given the recent increase in the prevalence of obesity and MetS, it is clear that on top of their unquestionable polygenic component, other environmental factors such as lack of physical activity or high caloric diets are likely contributors to their progressive acceleration. However, individual heterogeneity in genetic and environmental profiles makes the search for specific clues and mechanisms facilitating adipose tissue expansion and its related metabolic complications in a general population a daunting task. One suitable clinical study design setting to at least homogenize and eliminate the genetic component of this challenge is the twin design. Study of monozygotic (MZ) twin pairs, discordant for body weight, provides an opportunity to explore the initial effects of acquired obesity and related complications since these individuals share not only an identical genetic background at the DNA sequence level, but also early life events and family environment. Identification of mechanisms behind the traits related to acquired obesity and MetS is also relevant from the therapeutic point of view, as these mechanisms may point to targets for specific disease phenotypes that are not related to specific genetic makeups.

To obtain a global view of human adipose tissue lipidome in different degrees of acquired obesity, here we perform lipidomic analyses of adipose tissue in twin pairs discordant for obesity but still metabolically compensated. In parallel, we studied more evolved states of obesity by investigating a separate set of morbidly obese subjects. Information gathered from these experimental groups was used for molecular dynamics (MD) simulations of lipid bilayers using bioinformatics approaches, and the conclusions were further supported by in vitro adipocyte confirmatory studies. This approach uncovered the potential physiological mechanisms by which adipocyte membranes adapt to adipose tissue expansion associated with positive energy balance, ultimately leading to obesity. Furthermore, we demonstrate how in extreme obesity, failure of this adaptation is associated with the pathological metabolic manifestation of obesity.

## Results

### Twin Study Design

We first investigated acquired obesity independent of genetic influences in 13 MZ twin pairs discordant for body mass index (BMI), and nine BMI-concordant MZ twin pairs, of which five pairs were overweight (Table S1). The obese individuals were young adults and were considered healthy obese, i.e., with no clinical comorbidities, and thus in the early stages of the development of obesity. In this cohort, we have previously found that obese individuals, as compared to their healthy lean twins, already show signs of insulin resistance [10], exhibit a pro-inflammatory serum lipidomic profile [11], have diminished mitochondrial DNA copy number and dysregulated expression of mitochondrial pathways such as branched chain amino acid catabolism, and have elevated inflammatory and immune response pathways in the adipose tissue [12].

Analysis of the weight-discordant twin pairs showed that the obese twins were on average 15.2 kg (20%, 5.3 kg/m^2^) heavier than the non-obese twins and had more fat subcutaneously, intra-abdominally, and in the liver (Table S1). Average adipocyte diameter was 17% larger in the obese than in the non-obese twins, and, interestingly, there was also a very high degree of intra-pair similarity of fat cell size (FCS) (Figure S1). As expected, adiponectin levels were lower, and leptin and hsCRP levels higher, in the obese twins. The obese twins were also more insulin resistant, as evidenced by the lower M-value and higher fasting plasma glucose and serum insulin levels. Among the dietary intake variables, the proportional intake of polyunsaturated fatty acids (PUFAs) was 26% lower in heavy twins as compared to their lean counterparts (*p*<0.05). The analysis of weight-concordant twin pairs showed no intra-pair differences in FCS, adipokines, or insulin sensitivity (Table S1).

### Adipose Tissue Lipidome in Acquired Obesity

To characterize the adipose tissue lipidome in acquired obesity, we applied the global lipidomics approach using ultra performance liquid chromatography coupled to mass spectrometry (UPLC-MS). The analysis was performed in positive ion mode (ESI+), which is sensitive to neutral lipids (such as triacylglycerols) and major phospholipid classes such as phosphatidylcholines (PCs), phosphatidylethanolamines (PEs), and sphingomyelins. A total of 313 lipids were detected and quantified in each of the 44 samples analyzed. Additionally, free cholesterol was determined by gas chromatography coupled to mass spectrometry (GC-MS), and the data were included in the lipidomic dataset. Since we considered that adipocyte size might be a factor affecting membrane and cell lipid composition, we investigated the lipidome redistribution in relation to the cell size. Thus, following the calibration with internal standards we further normalized the data by the total amount of detected phospholipids in each sample. This allowed us to investigate the relative compositional changes of adipose tissue lipids.

**Figure 1:**
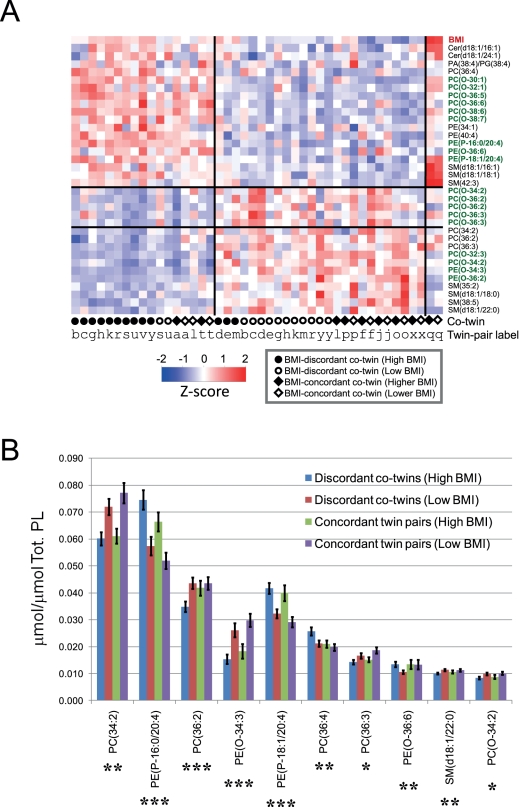
Adipose tissue lipidome in acquired obesity. Lipidomic analysis covered 314 molecular lipids in adipose tissue biopsies from 44 subjects (13 twin pairs discordant for BMI, nine twin pairs concordant for BMI). Thirty-four lipids were differentially regulated when comparing obese and lean weight-discordant twins (FDR *q*<0.05). (A) Lipidomic profiles of the 34 differentially regulated lipids in acquired obesity, shown for all subjects included in the study. The detected ether phospholipids are marked in green. The twin status for each subject is shown at the bottom. (B) Concentration, shown as fraction of the total phospholipid concentration as measured by lipidomics (Tot. PL), of the 11 most abundant differentially regulated lipids from (A), shown separately across the four groups corresponding to lean and heavy twins discordant for BMI, and the concordant twin pairs divided into two groups: high BMI (BMI≥25 kg/m^2^) and low BMI (BMI<25 kg/m^2^). *p*-Values are shown for pairwise *t* test comparison of discordant twins. *, *p<*0.05; **, *p<*0.01; ***, *p<*0.001. Error bars are ± SEM. SM, sphingomyelin.

When comparing the weight-discordant twins, lipidomic analysis revealed characteristic differences in cellular phospholipids (Figure 1A). The dominating characteristic of adipose tissue in the obese twins was the elevation of PUFA-containing phospholipids, which were predominantly ether lipids, and proportional diminishment of phospholipids containing shorter and more saturated fatty acids. These lipids were clustered according to BMI in both the discordant and concordant twin pairs, suggesting that these observed changes are characteristic of adipose tissue expansion per se, irrespective of genetic makeup. Figure 1B further illustrates this by showing the normalized concentrations of the most abundant significantly altered lipids. The most abundant PUFA-containing ether lipids were confirmed to be plasmalogens (Figure S2). Partial least squares regression of lipidomic data related to FCS showed that the changes in the top-ranking lipids associate with the increase of adipocyte cell size in obesity (Figure S3). In line with this, triacylglycerols were also elevated in the adipose tissue of obese twins at the marginal significance level false discovery rate (FDR) *q*<0.1 (Figure S4). Free cholesterol in adipose tissue did not differ significantly between the obese and lean twins (Figure S5).

### Acquired Obesity Leads to Selectively Enhanced Fatty Acid Desaturation and Elongation in Adipose Tissue

The observed changes in phospholipids in acquired obesity appear to be highly selective, showing both functional group as well as fatty acid specificity. It is known that PUFAs are selectively targeted to plasmalogens [13],[14], which could explain enrichment of adipose tissue ether lipids in acquired obesity (Figure 1). Notably, among the elevated plasmalogens observed in the obese individuals, the fatty acid dominantly esterified in the *sn*-2 position is arachidonic acid and not docosahexanoic acid (DHA), which is also commonly found in plasmalogens [13].

**Figure 2:**
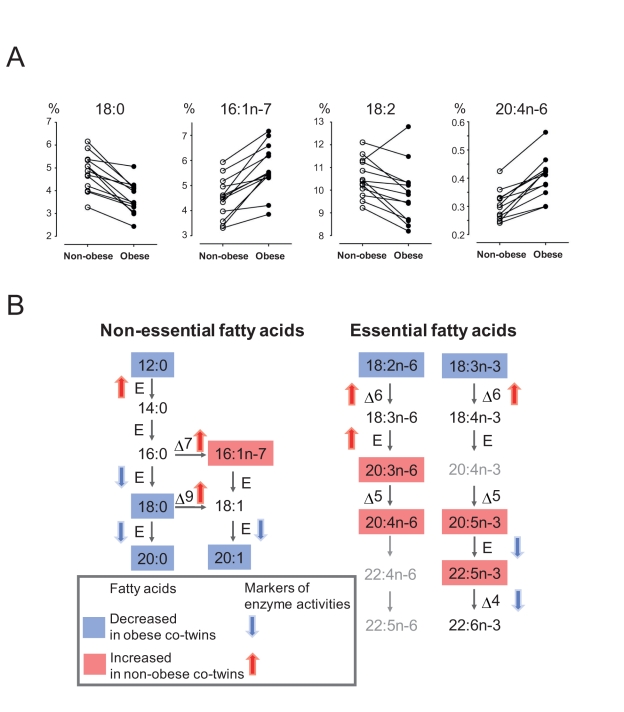
Fatty acid composition of adipose tissue in acquired obesity. Fatty acid profile was measured in adipose tissue biopsies in each of the 44 subjects. Complete results are shown in Table S2. (A) Selected fatty acid relative amounts in 13 twin pairs discordant for BMI. Lines connect the pairs of twins. (B) Schematic representation of fatty acid compositional changes when comparing heavy and lean obesity-discordant twins. Significant changes (*p*<0.05; pairwise *t* test) are color-coded. The activities of specific fatty acid elongation or desaturation steps are estimated by appropriate fatty acid concentration ratios.

To investigate whether specific global fatty acid compositional changes occur in the adipose tissue in response to weight gain, we analyzed fatty acid profiles in weight-discordant twin pairs. We found marked differences in several fatty acids (Table S2), which included diminishment of stearic (C18:0), linoleic (C18∶2n6), and α-linolenic (C18∶3n3) acids and elevation of palmitoleic (C16∶1n7) and arachidonic (C20∶4n6) acids (Figure 2A). In relation to known dietary intake, the data are consistent with diminished PUFA intake (decreased linoleic and α-linolenic acids), while other changes appear to reflect an induced specific program of fatty acid desaturation and elongation (Figure 2B), which leads to elevated palmitoleic acid via desaturation of palmitic acid, and to elevated long-chain PUFAs up to arachidonic acid, but not to DHA. The specific changes in PUFA composition are consistent with the observed changes in phospholipids. However, the elevation of esterified palmitoleic acid in the adipose tissue of the obese twins was not reflected in significantly elevated free palmitoleate levels in serum (Table S3 and Figure S6).

### Elevated PUFA-Containing Phospholipids Help Maintain Membrane Fluidity and Thickness

The observed remodeling of membrane phospholipids in expanding adipocytes of obese twins suggests that these changes may also have an effect on membrane properties such as membrane fluidity (packing), order, and thickness, which are known to affect cellular physiology [15],[16]. There is evidence from in vitro model systems and from atomic-scale MD simulations of lipid bilayers that an increase in PUFA content will increase membrane fluidity [17]–[19]. The lateral diffusion of lipids [20] and the permeability of the membrane to small molecules [21] are dependent on the fluidity through packing and degree of order in the bilayer. However, the effect of the vinyl-ether bond in plasmalogens on membrane fluidity is poorly understood and has not yet been modeled using MD simulations.

**Figure 3:**
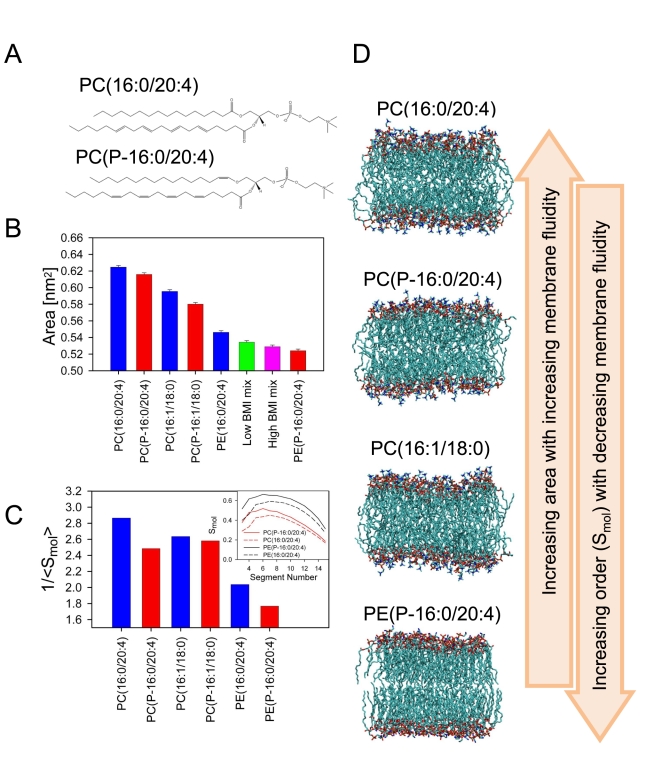
Large-scale molecular dynamics simulations of different lipid membrane systems. Six single-lipid component systems were studied, representing major groups of lipids found to be differentially regulated in acquired obesity (Figure 1A). Two lipid mixes, corresponding to observed concentration changes of most abundant lipids (Figure 1B), were also studied to represent lipid membrane composition in heavy and lean weight-discordant twins. (A) Structures of two PUFA-containing PCs included in the simulations, one plasmalogen and one ester-bonded. (B) Average area per lipid of the eight simulated bilayer systems, indicating the packing or fluidity of the bilayer (smaller area means decreased fluidity). (C) The molecular order parameter (*S*_mol_) profiles along selected unsaturated acyl chains. Segment number means the carbon position starting from ester-vinyl linkage and ending at the methyl group in the end of the chain. (D) Snapshots from the end of four simulations, with the lipids ordered from top to bottom according to decreased fluidity (area per lipid parameter from [B]).

To study the consequences of altered lipidomic profiles on membrane biophysical properties, we performed atomic-scale MD simulations of eight different membrane systems. Based on measured abundance of differentially regulated lipids (Figure 1), the following lipid types were used in simulations: PC(16∶1/18∶0), PC(P-16∶1/18∶0), PC(16∶0/20∶4), PC(P-16∶0/20∶4), PE(16∶0/20∶4), and PE(P-16∶0/20∶4). Structures of PC(16∶0/20∶4) and PC(P-16∶0/20∶4) are shown in Figure 3A. In addition to six one-component bilayers composed of these lipids individually, we also studied two additional mixtures of PC(16∶1/18∶0) and PE(P-16∶0/20∶4) with PE concentration of 59 mole percent (low BMI mix) and 70 mole percent (high BMI mix). The mixtures were designed to mimic the compositional difference between the obese and non-obese twins (Figure 1B). The selection of the specific six lipid molecules was motivated by the need to characterize specific effects due to three different types of structural changes observed in the lipids: (1) functional group (PE versus PC), (2) vinyl-ether versus ester bond in *sn*-1 position, and (3) degree of saturation in the *sn*-2 chain. There is reason to stress that considering all possible lipid combinations in membranes is not feasible because of the major computational cost. Therefore, the above choice of the eight model systems aims to clarify the overall effects on membrane fluidity arising from the three different structural changes observed in lipids, but with a reasonable cost. Yet the total simulation time is major, about 1 µs.

Figure 3B displays the average surface area per lipid, describing the overall packing of the lipids within the membrane plane. The larger the area per lipid, the more fluid the membrane is. Figure 3C shows that when plasmalogens are replaced with the corresponding ester lipids, the area per lipid increases by about 0.01–0.02 nm^2^. An increase of 0.07–0.09 nm^2^ is found when PE is replaced by PC headgroup. Finally, increasing the unsaturation of PC acyl chains increases the average surface area per lipid by 0.02–0.03 nm^2^. Together, membrane fluidity is promoted by decreasing plasmalogen concentration, using PC instead of PE headgroup, and increasing PUFA content. The snapshots in Figure 3D illustrate different packings and thus different degrees of fluidity in four of the studied bilayers.

As expected, membrane thickness is negatively correlated with area for all studied systems. Furthermore, increased lateral packing correlates with a higher conformational order of the acyl chains. To illustrate this point, Figure 3C shows the inverse of molecular order parameter (*S*_mol_) averaged over the saturated segments of the *sn*-1 acyl chains. It is clear that the *sn*-1 chains of plasmalogens are more ordered than the corresponding chains in the ester-bonded lipids.

To our surprise, no marked differences in the surface area and thus fluidity are observed when high and low BMI lipid mixtures are compared (Figure 3B). The data therefore suggest that the increased fluidity due to elevated PUFA content in membrane phospholipids is compensated for by decreased fluidity of the elevated PE plasmalogen lipids, with the final result that there is no change in membrane fluidity and thickness. The membrane clearly has compensatory mechanisms to maintain its fluid nature.

### Adipose Tissue Network

Next, we investigated the regulatory mechanisms that may be behind the observed lipid remodeling. Because of the intrinsic complexity of lipid metabolism, interactions of multiple components are likely involved in the regulation of lipid changes [22]. To capture such functional interconnections between the biological entities, we considered a network-based approach to be more suitable than studies of differential expression changes at the individual gene level. We selected ten clinical variables, 31 lipid-metabolism-related genes from the published dataset [12] obtained from the same samples used in the present study, two pathway profiles reflecting major changes observed in pathway analysis [12], and ten lipid variables representative of major changes observed in adipose tissue lipid profiles (Table S4). As the only “input” variable, we used dietary PUFA intake (PUFA percent). To distinguish direct and indirect interactions of these variables, we utilized the QPGRAPH method, which has been previously applied to study gene regulatory networks based on microarray data [23]. The key idea of QPGRAPH is to use partial correlations as a measure of dependency and build an undirected Gaussian graphical model where the variables are connected if and only if their partial correlation is significantly non-zero. Unlike the pairwise measure of associations, e.g., Pearson correlation coefficients, partial correlation provides a stronger criterion for dependency by adjusting for confounding effects, and thus removes spurious associations to a large extent. This is particularly favorable for such an integration of multiple layers of information, as it inherently filters out false positives by discovering only those direct interactions with high confidence.

**Figure 4:**
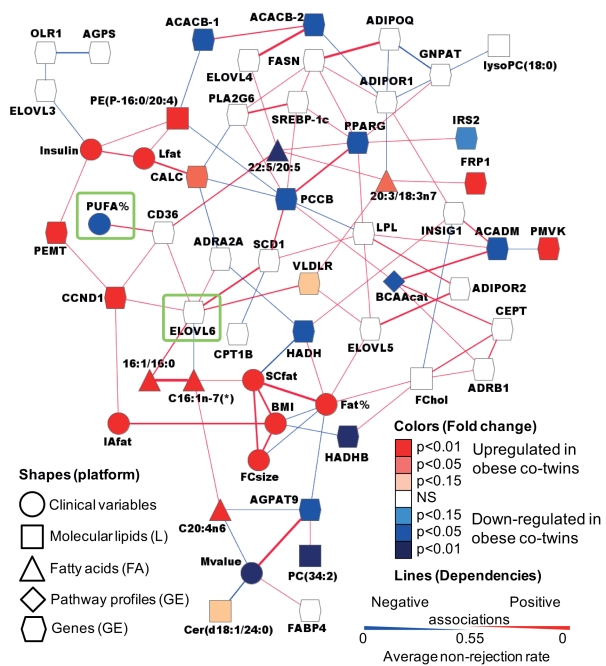
Regulation of lipid remodeling in adipose tissue. A dependency network was constructed from selected gene expression, clinical, and lipidomic data from twin pairs discordant for BMI. Node shapes represent different types of variables and platforms (L, UPLC-MS lipidomics; FA, fatty acid gas chromatography; GE, gene expression), node color corresponds to significance and direction of regulation (full data in Table S4), and line width is proportional to strength of dependency. The variables are connected by an edge if and only if their partial correlation is significantly non-zero. PUFA percent and the nearest network hub (Elovl6) are highlighted with green squares. The cutoff for the presence of edge was set at 0.55 by the average non-rejection rate, i.e., an edge in the graph tested positive in 55% of the 500 samplings.

The data-driven dependency network in twins discordant for obesity is shown in Figure 4. Notably, PUFA percent was connected to CD36, an important fatty acid transporter [24], which was further connected to fatty acid elongase Elovl6. In such a network context, identification of genes connected to many other genes or variables of interest, i.e., so-called network hubs, is of particular interest. For example, despite not being differentially regulated itself, Elovl6 appears to be an important hub, with seven connections, including desaturase SCD1. Among the other important regulators of lipogenesis, PPARγ was significantly down-regulated in obese twins, and, along with fatty acid elongase Elovl4, which was not differentially regulated, was associated with the decreased fatty acid ratio of C22:5 versus C20:5, a step which appears to affect the balance between the amounts of arachidonic acid and DHA (Figure 2). In agreement with earlier findings [25], the decreased expression of PPARγ was also associated with decreased expression levels of insulin receptor substrate 2 (IRS2).

### Elovl6 Regulates the Membrane Phospholipid Remodeling in Adipose Tissue

**Figure 5:**
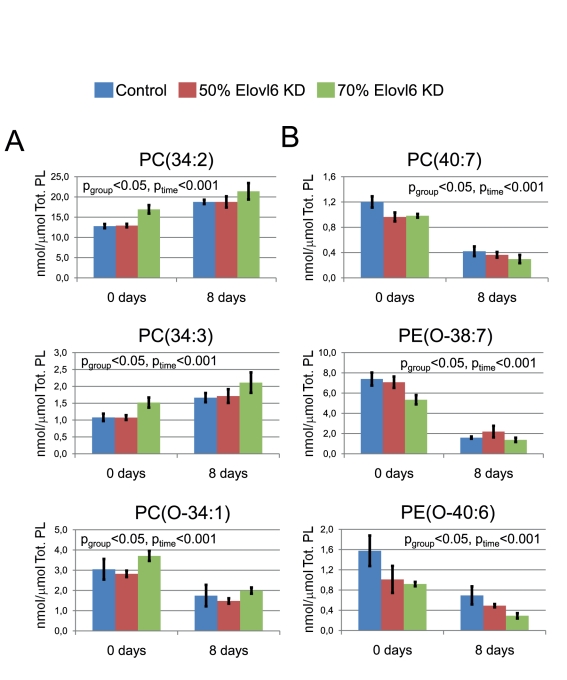
Regulation of lipidomic profiles by Elovl6 in differentiated adipocytes. 3T3-L1 preadipocytes were differentiated in wild-type (control) cell line, and following 50% and 70% Elovl6 knockdown (KD) (three biological replicates for each group). Lipidomic analysis was performed in preadipocytes and in mature adipocytes. One-way ANOVA was performed to test for the significance of time (mature adipocytes versus preadipocytes) and group-specific lipid changes. Error bars are ± SEM. Tot. PL, total phospholipids as measured by lipidomics. (A) The upregulated phospholipids in Elovl6 knockdown cells are mainly shorter chain and saturated. Selected lipids are shown. (B) The diminished phospholipids are mainly PUFA containing, predominantly ether lipids. Selected lipids are shown. All differentially regulated phospholipids in Elovl6 knockdown cells are listed in Table S5.

We then investigated whether the observed network might be amenable to modulation of the lipid profiles observed in the weight-discordant twins. Given its position as a network hub (Figure 5) and its known regulatory role in the control of cellular fatty acid composition [26], we hypothesized that ablation of Elovl6 might be an upstream regulator of the lipid remodeling observed in obese twins, sensitive to dietary stimulus such as relative decrease in PUFA intake. To gain further insights we hypothesized that ablation of Elovl6 in the 3T3-L1 adipocyte cell line might reveal a mirroring lipid pattern. In fact, lipidomic analysis of preadipocytes and mature adipocytes revealed that ablation of Elovl6 leads to a lipid profile opposite to the one observed in obese twins, thus supporting our model (Figure 5 and Table S5). Specifically, knock-down of Elovl6 leads to a reduction in PUFA-containing phospholipids, which are predominantly ether-bonded, and to a proportional elevation of shorter and more saturated phospholipid species.

### Membrane Lipid Remodeling Breaks Down in Morbid Obesity

**Figure 6:**
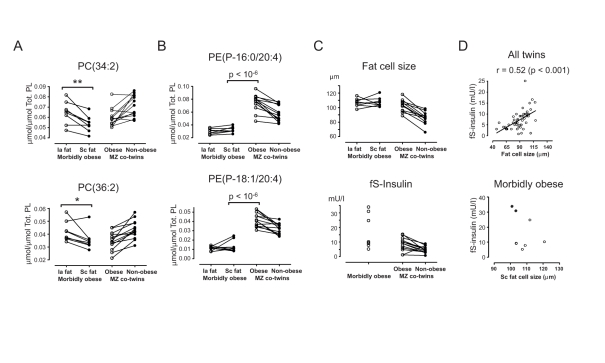
Adipose tissue in morbid obesity is characterized by diminished levels of PUFA-containing ether lipids. Comparison of the amounts of abundant phospholipids differentially regulated in acquired obesity in the subcutaneous adipose tissue (Figure 1B), shown as a fraction of the total phospholipid concentration, with the mean amounts in intra-abdominal (Ia fat) and subcutaneous (Sc fat) tissue biopsies of eight morbidly obese subjects from an earlier study [27]. Tot. PL, total phospholipids as measured by lipidomics. (A) There are no marked differences for abundant shorter-chain and saturated phospholipids. *, *p<*0.05; **, *p<*0.01. (B) The most abundant ether phospholipids are markedly diminished in morbid obesity. (C) FCS and fasting serum insulin (fS-Insulin) in morbidly obese subjects and twins discordant for obesity. (D) FCS correlates with fasting serum insulin in twins (all 44 subjects included in the study), but not in morbidly obese subjects. The two subjects diagnosed with T2D are marked with filled circles.

To investigate the lipid profile in pathogenic stages of obesity, we compared the adipose tissue lipid compositional changes in obesity-discordant twin pairs (Figure 1B) with lipidomic profiles from our recent study of eight morbidly obese subjects recruited among patients undergoing laparoscopic surgery for the treatment of obesity [27]. The BMI range was 47.0–60.4 kg/m^2^; four subjects had elevated fasting serum insulin (>10 mU/l), and among these two subjects were diagnosed with T2D. The levels of shorter and more saturated phospholipids were similar in the morbidly obese subjects and healthy twin pairs (Figure 6A). However, the proportion of PUFA-containing ether lipids was markedly lower (Figure 6B), indicating that the mechanism of lipid remodeling observed in healthy obese subjects had broken down or that it was unable to compensate at that level of stress. In contrast to the obese twins, the linear dependence of fasting serum insulin on FCS appears to have broken down in the morbidly obese subjects (Figure 6C and 6D). The major outliers were the two subjects diagnosed with T2D, who had the smallest FCS and the highest fasting serum insulin concentrations (Figure 6D).

## Discussion

Adipose tissue is a highly active endocrinometabolic organ whose main role is to provide efficient storage and mobilization of lipids to fulfill bioenergetic demands. Adipocyte function depends on the homeostasis of important cellular lipid mediators and lipid structural components of biological membranes required for accurate functional responses. These lipid functions are interconnected, e.g., the membrane lipids also serve as precursors of lipid mediators. Modern lipidomics technologies enable characterization of cellular lipidomes across multiple structural and functional groups. However, the analysis and interpretation of data from such datasets are commonly limited to the level of biochemical and signaling pathways. In this study, we presented a new approach to study global lipidomes in tissues and cells by combining cellular lipid networks with lipid membrane modeling. This strategy enabled us to identify adaptive mechanisms that may lay behind the characteristic remodeling of the adipose tissue lipidome in response to positive-energy-balance-induced adipose tissue expansion during the evolution of obesity.

There is evidence suggesting that the total number of adipocytes in humans tends to remain constant during a lifetime [28] under physiological conditions. Consequently the excess lipid load in acquired obesity poses an extra layer of stress on the adipose tissue, which can be compensated by increasing the size of the adipocytes and/or overstimulating the normal processes of adipocyte turnover in the adipose tissue. This relative stability of the adipose tissue cellular composition requires active regulatory mechanisms. In agreement with this perspective, our data show that FCS is increased in obese individuals relative to their lean twins in weight-discordant MZ twin pairs (Figures 6C and S1). We also observed a high degree of intra-pair similarity of FCS in weight-concordant MZ twin pairs (Figure S1), which suggests a strong genetic component tightly regulating FCS. These changes in cell size may lead to and/or be affected by minor variations in membrane composition, resulting in changes in membrane fluidity and lateral pressure [9]. It would be expected that these changes would consequently affect the membrane protein function and thus cellular physiology. In this context we consider adipose tissue expansion in response to positive energy balance to be a challenge for the maintenance of membrane integrity and function, requiring potent adaptive allostatic responses.

With adipose cell expansion, more phospholipids have to be incorporated into the cellular membranes. However, this process also requires a very accurate quality control system that ensures that irrespective of the available lipid pool, the composition of the membrane and its functionality is appropriate in expanding adipocytes and newly recruited adipocytes. We found that the expansion of adipose tissue is accompanied by a proportional increase of PUFA-containing ether lipids and a decrease of more saturated and shorter-chain ester-bound lipids (Figure 1). These changes were also reflected in the altered fatty acid composition (Figure 2). Given that no increase was observed in saturated short-chain fatty acids (Figure 2), it is unlikely that de novo fatty acid synthesis could explain the observed fatty acid profile. Of interest, proportional dietary intake of PUFA was lower in obese twins than in their lean counterparts, and this was reflected in the diminished linoleic and α-linolenic acids (Figure 2). However, paradoxically, the concentration of arachidonic acid was increased in the obese twins, as was also reflected in the altered profile of the phospholipids. Notably, this increase was specific to arachidonic acid since there was no difference between the levels of DHA of the obese and lean twins (Figure 2). The observed lipid changes are not systemic since we have previously shown that the ether lipids are down-regulated in the serum of the obese twins [11].

**Figure 7:**
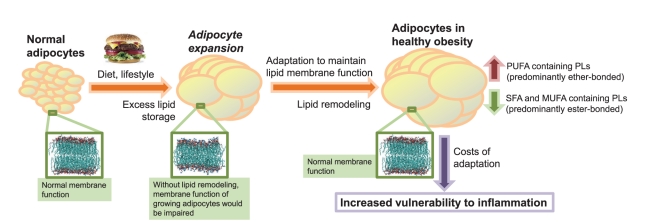
Model of physiological regulation of lipid membrane composition in obesity. MUFA, monounsaturated fatty acid; PL, phospholipid; SFA, saturated fatty acid.

Our findings support the view that positive energy balance leading to obesity initiates a program of membrane lipid remodeling in the adipose tissue, involving increased biosynthesis of unsaturated fatty acids including arachidonic acid (Figure 7). In agreement with earlier findings [29]–[31], arachidonic acid is actively incorporated into the membrane phospholipids and selectively targeted to ethanolamine plasmalogens (Figure 1). However, if the only membrane compositional changes were these specific changes in the fatty acid composition, the membrane would become more fluid [19]. Instead, our membrane simulations showed that the targeting of PUFAs to ether lipids, and preferentially to the most abundant ethanolamine plasmalogen, helps to maintain membrane biophysical properties. In fact, it is known that a relative increase in the PE/PC ratio contributes to increasing the rigidity of the membrane [32] and therefore compensating for increased fluidity mediated by unsaturated fatty acids. Here we also provided evidence based on biophysical modeling that supports the view that the presence of a vinyl-ether bond in the *sn*-1 position of phospholipids contributes to further stiffening of the membrane.

The adaptation of adipose tissue membranes in obesity may carry a price. In fact, plasmalogens can serve as antioxidants against reactive oxygen species and might thus protect the cells from oxidative stress [33],[34]. However, when arachidonic-acid-containing plasmalogens are under oxidative stress, they become precursors of arachidonic-derived lipid mediators such as leukotrienes and hydroxyeicosatetraenoic acids [29],[31],[35],[36]. These reactive lipids are important mediators of inflammatory response [37]. Thus, this remodeling process occurring in the membranes may make the adipocytes more vulnerable and prone to inflammatory responses (Figure 7), in agreement with the elevation of inflammatory pathways in the obese twins of weight-discordant twin pairs [12]. In the context of inflammation, a crucial step in the observed lipid remodeling is the relative decrease of C22:6 (DHA) and C22:5 as compared to C20:5, leading to a relative increase of arachidonic acid content relative to DHA in acquired obesity (Figure 2). In contrast to arachidonic-acid-derived lipid mediators, the DHA-derived lipid mediators tend to have anti-inflammatory properties [38]. We propose that stimulation of fatty acid elongation from C20:5 to C22:5 and desaturation from C22:5 to C22:6 could be one strategy to maintain the membrane function in acquired obesity, while also avoiding the collateral damage of increasing the vulnerability of adipose tissue to inflammation.

We observed a high degree of linearity in FCS variation in weight-discordant twin pairs (Figure S1). This suggests that adipocytes of these relatively modestly obese twins are still within the range of normal expansion and meeting the demands imposed by their specific nutritional demands. However, this physiological process appears to be disrupted in states of excessive adipose expansion beyond homeostatic capacity in morbidly obese subjects, characterized by decreased concentrations of ethanolamine plasmalogens compared to the “healthy obese” twins (Figure 6B). In morbidly obese individuals, fasting serum insulin does not correlate with FCS (Figure 6D). The two diabetic subjects in this group had, in fact, the smallest FCS, indicating that the limit of adipocyte expandability may have been reached. Identification of pathways involved in regulation of early adaptation to excess lipid load is important, since these pathways might provide clues about prediction, prevention, or treatment of obesity-related metabolic complications.

We used the network approach, rather than a simpler gene expression level analysis, to determine critical nodes that could control adipose tissue lipidome remodeling. Doing so, we detected Elovl6, which, while not changed on an mRNA expression level itself, appeared to be connected to multiple different factors involved in controlling adipose tissue lipidome (Figure 4). To validate that perturbations in Elovl6 function were capable of mediating at least some of the changes observed in the obese twins, we used a cell line to validate our network. Consistent with our hypothesis, knockdown of Elovl6 in 3T3-L1 adipocytes mirrored the results found in the obese twins, therefore suggesting that the observed lipid remodeling may be amenable to genetic modulation.

As a potential limitation of the study, lipidomic analysis was performed in adipose tissue biopsies and not in isolated adipocytes. It is known that obesity is associated with the infiltration of inflammatory cells in the adipose tissue [39],[40]. We have in fact observed elevation of multiple inflammatory and immune pathways in the obese twins in our sample [12]. However, adipocytes and their lipids constitute most of the adipose tissue pool per unit volume, and it is therefore unlikely that the observed large change in phospholipid composition is due to changes in percent cellular composition of the adipose tissue. Furthermore, if that were the case, the lipidome changes observed in morbidly obese subjects (Figure 6) would have followed the same trend, but to an even greater extent, as observed in the obese twins.

One of the advantages of the twin study setting in the context of understanding human obesity is the possibility to explore the initial effects of acquired obesity and related complications independent of genetic makeup. The weight differences between the twins in the discordant pairs began to emerge at 18 y of age [41], and diet appears to be the major factor behind these differences [42]. The discordant pairs were of the same age (mean ± standard deviation  = 25.6±1.2 y) as the concordant pairs (25.7±1.2 y); therefore, age cannot be considered a confounding factor in our study. The fact that it was extremely challenging to find even this number of MZ twin pairs discordant for weight (only 14 pairs out of the cohort of 2,453 twins born in Finland in the years 1975–1979, among which adipose tissue was available from 13 pairs) and the fact that the obesity phenotypes found were within a narrow range, suggest a strong genetic and early environmental basis for susceptibility to obesity. Therefore, our study design offers considerable advantages over other types of studies in humans where groups with different obesity phenotypes also differ for their genotypes.

In summary, we have used a novel approach to study cellular lipidomes, and our study proposes an allostatic mechanism by which normal membrane function is maintained in the expanding adipose tissue at the expense of increasing its vulnerability to inflammation. The lipid remodeling seems to be triggered by the proportional decrease of PUFA content and is controlled by a complex network involving fatty acid desaturation and elongation. If small molecules could modulate this network for both membrane functional maintenance and vulnerability to inflammation, new opportunities may arise for the prevention or treatment of obesity-related metabolic complications.

## Materials and Methods

### Subjects and Twin Study Design

Twin pairs included in the current study were recruited from a population-based longitudinal study of five consecutive birth cohorts in Finland (1975–1979) of twins (*n = *2,453) [43], based on their responses to questions on weight and height at age 23–27 y. From this cohort, we searched for the top 5% most obesity-discordant MZ twin pairs (one twin non-obese [BMI ∼25 kg/m^2^] and the other one obese [BMI ∼30 kg/m^2^]), with no significant height differences (<3 cm). After screening all MZ twin pairs (*n = *658), we identified 18 pairs above the 95^th^ percentile of BMI differences (3.1 kg/m^2^) [10],[41],[44]–[47]. Fourteen of these pairs (eight male and six female pairs) were willing to participate, and thirteen pairs (eight male and five female pairs; BMI differences 3.3–10.0 kg/m^2^) had adipose tissue samples available for the present study. We also studied nine randomly selected weight-concordant MZ pairs (two male and three female overweight pairs and two male and two female normal-weight pairs, BMI differences 0.0–2.3 kg/m^2^). All pairs were Caucasian, and the discordant pairs were of the same age (mean ± standard deviation 25.6±1.2 y) as the concordant pairs (25.7±1.2 y). The subjects were healthy (based on medical history, clinical examination, and structured psychiatric interview), normotensive, and did not use any medications except contraceptives. Their weight had been stable for at least 3 mo prior to the study. Females were scheduled to attend during the follicular phase of their menstrual cycle. Monozygosity was confirmed by the genotyping of ten informative genetic markers [41]. Dietary information was collected based on 3-d food diary as described previously [10]. Further details on experimental methods are provided in Text S1.

The subjects provided written informed consent. A small amount of subcutaneous fat tissue aspirated by needle biopsy was used for the metabolic studies of adipose tissue, as described in the consent form. The protocol was designed and performed according to the principles of the Helsinki Declaration and was approved by the Ethical Committee of the Helsinki University Central Hospital.

### Culture, Differentiation, and Infection of 3T3-L1 Preadipocytes

3T3-L1 cells were cultured, and differentiated into adipocytes using the following protocol. 3T3-L1 preadipocytes were passaged in 25 mM glucose DMEM supplemented with 1% Pen-Strep (Sigma Aldrich P0781) and 1% glutamine (Sigma Aldrich G7513). Differentiation cells were plated into six-well culture dishes and allowed to grow to confluence. Two days post confluence 3T3-L1 preadipocytes were induced to become adipocytes. The induction medium was 25 mM DMEM with 10% fetal bovine serum (Sigma Aldrich), 1% L-glutamine, and 1% Pen-Strep (FBS medium) supplemented with 100 nM insulin, 1 µM dexamethasone, and 0.5 mM IBMX. At day 2 the medium was replaced with FBS medium with insulin alone. From day 4 until day 8 cells were grown in FBS medium alone until adipogenesis was complete. Differentiated cells were used only when at least 95% of the cells showed an adipocyte phenotype by accumulation of lipid droplets by day 8. Cells were analyzed by phase contrast microscopy. Elovl6 knockdown cells (or controls) were generated with the pSiren-RetroQ retroviral vector system. All retrovirally transfected 3T3-L1 cell lines were kept in puromycin-containing medium throughout culture and differentiation procedures.

### Retroviral Short Hairpin RNA Constructs for Elovl6

Stable knockdown of Elovl6 was achieved by expression of short hairpin RNA from the pSiren-RetroQ vector (Clontech). Target sequences for knockdown of Elovl6 (GenBank Accession number NM_130450) were identified using the Dharmacon siDesign center, and control cells were infected with virus for a scrambled short hairpin RNA. Custom oligonucleotides (sequences available on request) were designed to incorporate these sequences into short hairpin RNA expression sequence, and cloned into pSiren-RetroQ according to the manufacturer's instructions. The knockdown was confirmed at mRNA and enzyme activity levels (Figure S7) using the methods described in Text S2.

### Lipidomic Analysis of Adipose Tissue and 3T3-L1 Adipocytes

Global lipidomic profiles of adipose tissue biopsies were determined by using a UPLC-MS platform [48]. Data processing was performed using the MZmine software [49],[50]. Slightly different analytical methods were applied for the tissue biopsies and 3T3-T1 adipocytes (Text S3). Serum-free fatty acids as well as adipose tissue esterified fatty acids were measured by gas chromatography. Free cholesterol in adipose tissue biopsies was determined using GC-MS. Further details on lipidomic analysis are provided in Text S3.

### Statistical Methods

Statistical analyses were performed using the freely available R statistical software (http://www.r-project.org). FDR *q*-values [51] were computed using statistical methods from R package “qvalue.” Chemometric modeling using partial least squares [52] regression was performed using Matlab version 7.0 (Mathworks) and PLS Toolbox version 4.0 of the Matlab package (Eigenvector Research). The model-based clustering was performed using the MCLUST method [53], implemented in R (Text S4). Construction of the adipose tissue network for selected variables was performed using undirected Gaussian graphical Markov networks that represent *q*-order partial correlations between variables, implemented in the R package “qpgraph” [23] that forms part of the Bioconductor project (http://www.bioconductor.org). In these networks, missing edges denote zero partial correlations between pairs of variables, and thus imply the conditional independence relationships in the Gaussian case (Text S4). The network was visualized using Cytoscape [54] and yED graphical editor [55].

### Lipid Bilayer Simulations

All simulated bilayer systems consisted of 128 lipid molecules and about 3,500 water molecules. The initial structures of all bilayers were obtained by modification of a dipalmitoylphosphatidylcholine (DLPC) bilayer simulated for 130 ns as described in our previous study [56]. All the simulations were performed using GROMACS software package version 4.0.4 [57] over a time scale of 100 ns. The first 40 ns were considered an equilibration period, and the remaining period of 60 ns of each trajectory was analyzed. Further detail on experimental methods is provided in Text S4.

## Supporting Information

Figure S1 **Fat cell size in twins discordant and concordant for obesity.**
(0.06 MB PDF)

Figure S2 **ESI+ tandem mass spectrometry spectra of plasmalogens.** The plasmalogen identification using tandem mass spectrometry is based on characteristic peaks acquired in positive ion mode.
(0.03 MB PDF)

Figure S3 **Fat cell size in acquired obesity correlates with changes in phospholipid profile.** Partial least squares regression of 34 differentially regulated lipids (Figure 1A) on FCS. Each lipid and FCS variable *X* was twin-normalized using the formula *X*_norm_ = log_2_(*X*_heavy_/*X*_lean_), where *X*_heavy_ is the variable *X* value for the heavy twin and *X*_lean_ is the variable *X* value for the lean twin.
(0.01 MB PDF)

Figure S4 **Most abundant triacylglycerols in adipose tissue, sorted according to abundance in the lean twins of weight-discordant pairs.** When comparing obesity-discordant twin pairs (heavy versus lean twin) using pairwise *t* test, none of the shown triglycerides reached FDR *q<*0.05, but they were all marginally significant at FDR *q<*0.1. Error bars are ± standard error of the mean (SEM). Tot. PL, total phospholipids as measured by lipidomics.
(0.07 MB PDF)

Figure S5 **Free cholesterol in adipose tissue.** None of the comparisons are statistically significant. Error bars are ± SEM.
(0.02 MB PDF)

Figure S6 **Serum palmitoleate correlation with adipose tissue esterified palmitoleate.** Twin normalization was performed as described in Figure S3.
(0.03 MB PDF)

Figure S7 **Elovl6 enzyme activity and mRNA expression in 3T3-L1 cell lines.** (A) Elovl6 3T3-L1 knockdown cell lines have a functional reduction in C16:0–C18:0 fatty acid elongation ability. Elongation activity expressed as incorporation of radioactive malonyl-CoA into lipid fraction in picomoles per milligram of protein of isolated microsomes per minute. Palmitoyl-CoA was used as the substrate for elongation in the reaction. (B) Degree of knockdown of Elovl6 mRNA in 3T3-L1 cell lines. Expression in arbitrary units normalized to 18s housekeeping gene. *, *p*<0.05 versus control line, Student's *t* test followed by Bonferroni correction for multiple tests; *n = *3 replicates per group.
(0.04 MB PDF)

Table S1 **Physical and biochemical characteristics of weight-discordant (** ***n =*** **13) and weight-concordant (** ***n =*** **9) monozygotic twin pairs.** Data are median (interquartile range). ^a^Obese versus non-obese twins, paired Wilcoxon's test. ^b^ *n = *9 discordant pairs.
(0.04 MB DOC)

Table S2 **Fatty acid composition in adipose tissue lipids of weight-discordant (** ***n =*** **13) and weight-concordant (** ***n =*** **9) monozygotic twin pairs.** Data are median (interquartile range) (in molar precentage). ^a^Obese versus non-obese twins, paired *t* test.
(0.04 MB DOC)

Table S3 **Serum-free fatty acid composition in adipose tissue lipids of weight-discordant (** ***n =*** **13) and weight-concordant (** ***n =*** **9) monozygotic twin pairs.** Data are median (interquartile range) (in µmol/l). ^a^Obese versus non-obese twins, paired *t* test.
(0.04 MB DOC)

Table S4 **Selected variables included in the dependency network analysis.**
(0.10 MB DOC)

Table S5 **Top-ranking phospholipids (PC and PE class) differentiating mature adipocytes in Elovl6 70% knockdown cell line as compared to controls.**
(0.04 MB DOC)

Text S1 **Experimental methods in the twin study.**
(0.04 MB DOC)

Text S2 **Characerization of Elovl6 knockdown in vitro.**
(0.04 MB DOC)

Text S3 **Methods for lipidomic analysis.**
(0.05 MB DOC)

Text S4 **Computational and statistical methods.**
(0.06 MB DOC)

## Abbreviations

BMI: body mass index
DHA: docosahexanoic acid
FCS: fat cell size
FDR: false discovery rate
GC-MS: gas chromatography coupled to mass spectrometry
MD: molecular dynamics
MetS: metabolic syndrome
MZ: monozygotic
PC: phosphatidylcholine
PE: phosphatidylethanolamine
PUFA: polyunsaturated fatty acid
SEM: standard error of the mean
T2D: type 2 diabetes
UPLC-MS: ultra performance liquid chromatography coupled to mass spectrometry
