# Supplementary figures and images for: Association of Lipidome Remodeling in the Adipocyte Membrane with Acquired Obesity in Humans
Source: PLoS Biol. 2011 Jun 7;9(6):e1000623. doi: 10.1371/journal.pbio.1000623 (PMC3110175; doi:10.1371/journal.pbio.1000623)

## Fat cell size in MZ twins, TwinA vs TwinB

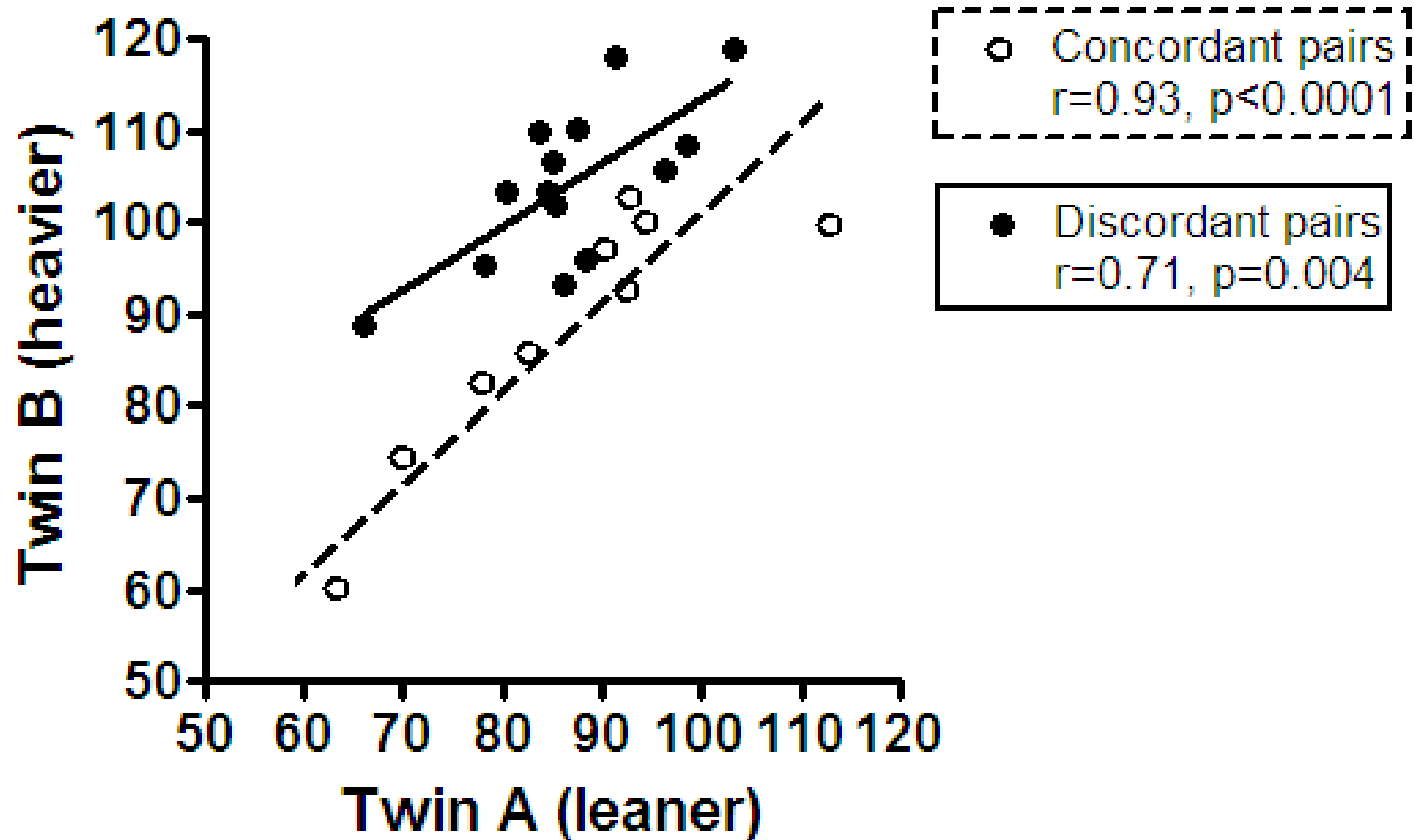

Supplement: Figure S1 — Fat cell size in twins discordant and concordant for obesity. (0.06 MB PDF) [file pbio.1000623.s001.pdf]

PE(O-36:5), confirmed as PE(P-16:0/20:4)

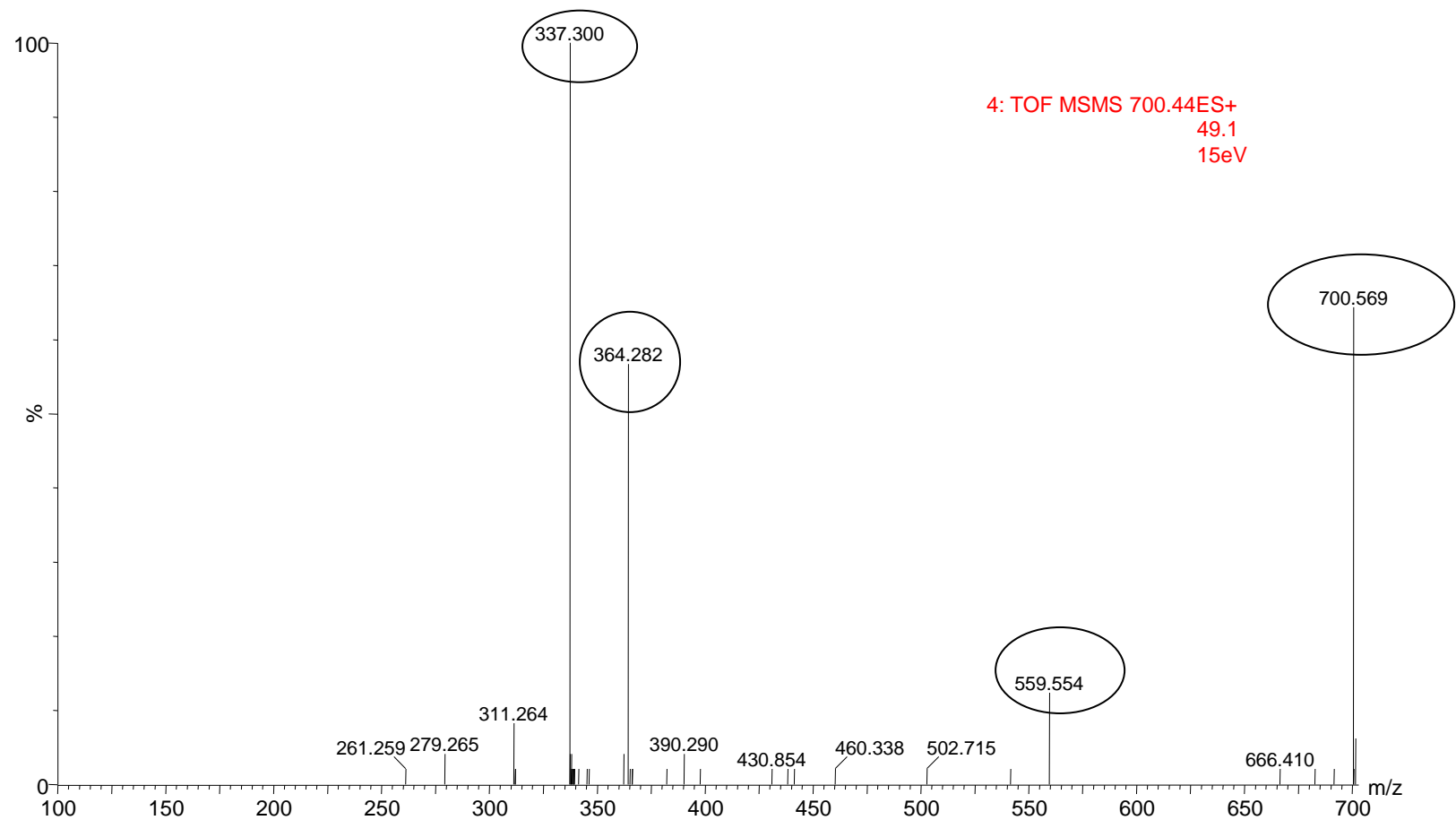

PE(O-38:6), confirmed as PE(P-18:1/20:4)

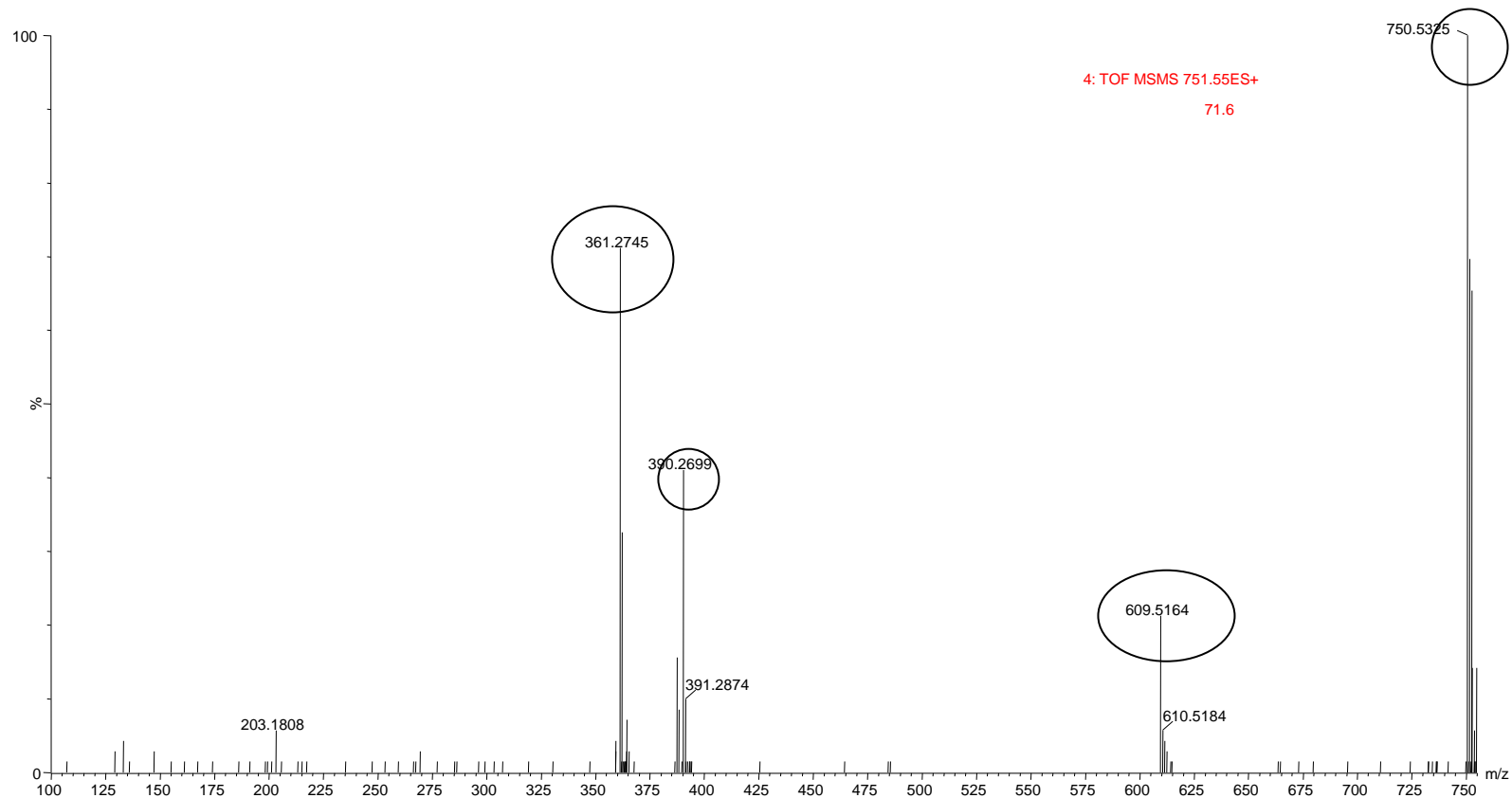

Supplement: Figure S2 — ESI+ tandem mass spectrometry spectra of plasmalogens. The plasmalogen identification using tandem mass spectrometry is based on characteristic peaks acquired in positive ion mode. (0.03 MB PDF) [file pbio.1000623.s002.pdf]

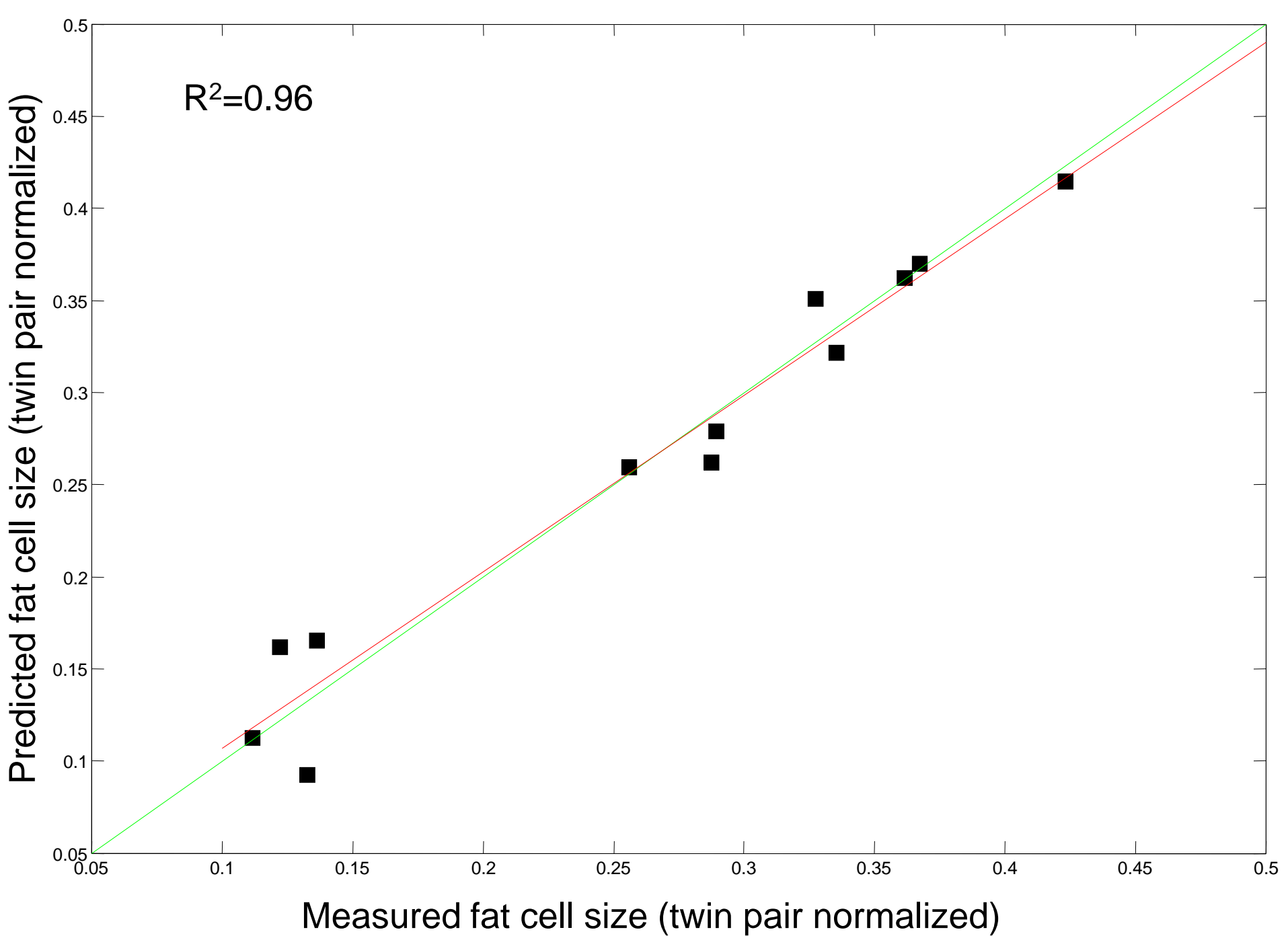

Supplement: Figure S3 — Fat cell size in acquired obesity correlates with changes in phospholipid profile. Partial least squares regression of 34 differentially regulated lipids (Figure 1A) on FCS. Each lipid and FCS variable X was twin-normalized using the formula X norm = log2(X heavy/X lean), where X heavy is the variable X value for the heavy twin and X lean is the variable X value for the lean twin. (0.01 MB PDF) [file pbio.1000623.s003.pdf]

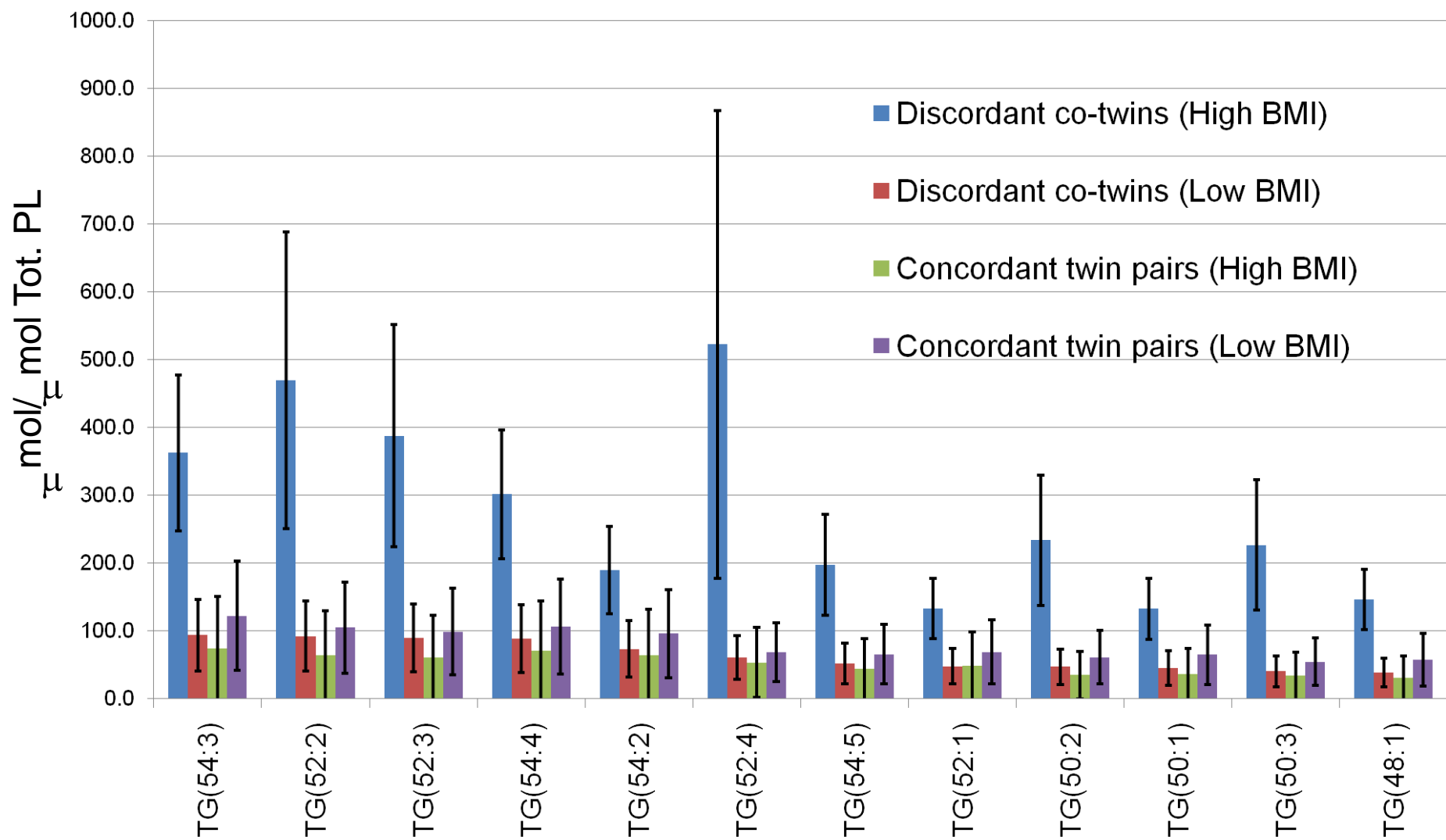

Supplement: Figure S4 — Most abundant triacylglycerols in adipose tissue, sorted according to abundance in the lean twins of weight-discordant pairs. When comparing obesity-discordant twin pairs (heavy versus lean twin) using pairwise t test, none of the shown triglycerides reached FDR q<0.05, but they were all marginally significant at FDR q<0.1. Error bars are ± standard error of the mean (SEM). Tot. PL, total phospholipids as measured by lipidomics. (0.07 MB PDF) [file pbio.1000623.s004.pdf]

## Free cholesterol

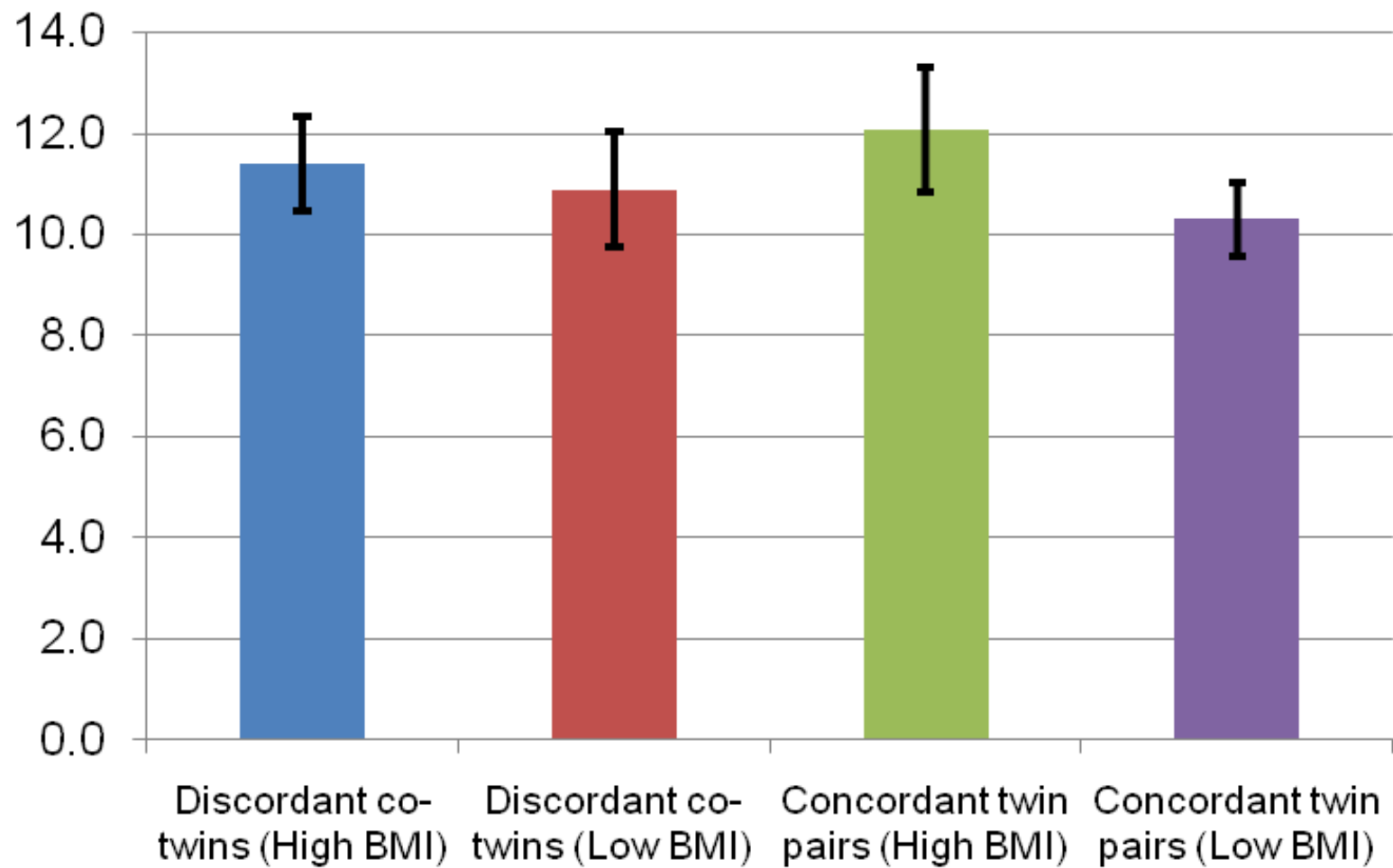

Supplement: Figure S5 — Free cholesterol in adipose tissue. None of the comparisons are statistically significant. Error bars are ± SEM. (0.02 MB PDF) [file pbio.1000623.s005.pdf]

- Weight-discordant twins
- Weight-concordant twins

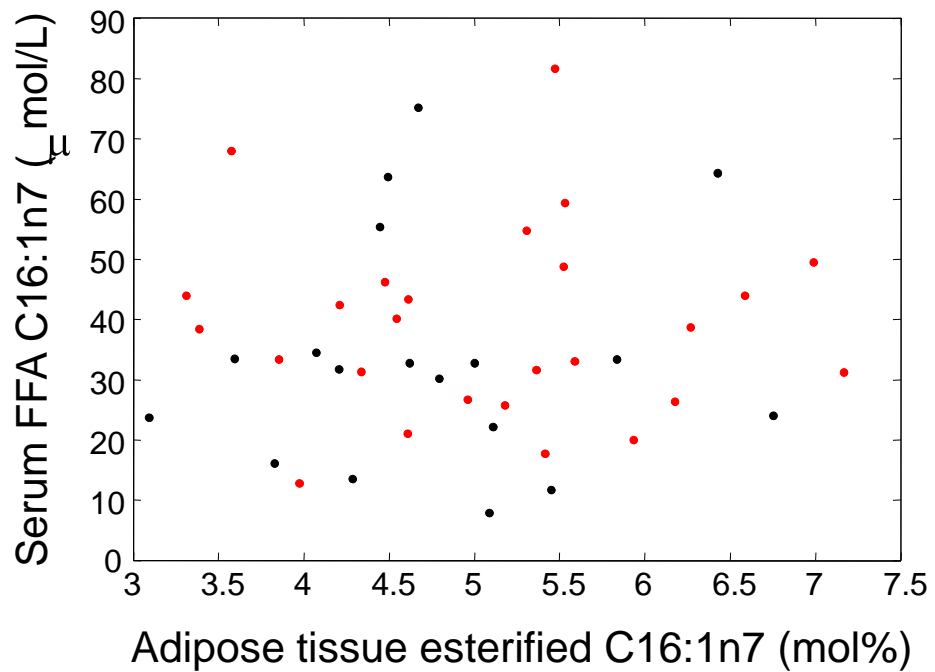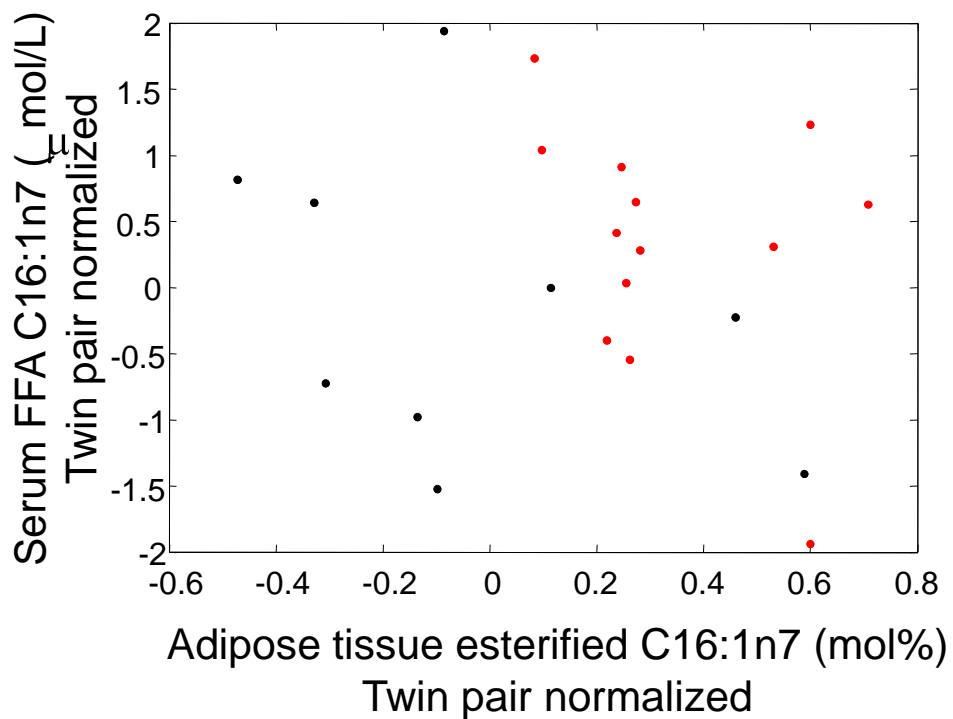

Supplement: Figure S6 — Serum palmitoleate correlation with adipose tissue esterified palmitoleate. Twin normalization was performed as described in Figure S3. (0.03 MB PDF) [file pbio.1000623.s006.pdf]

# A

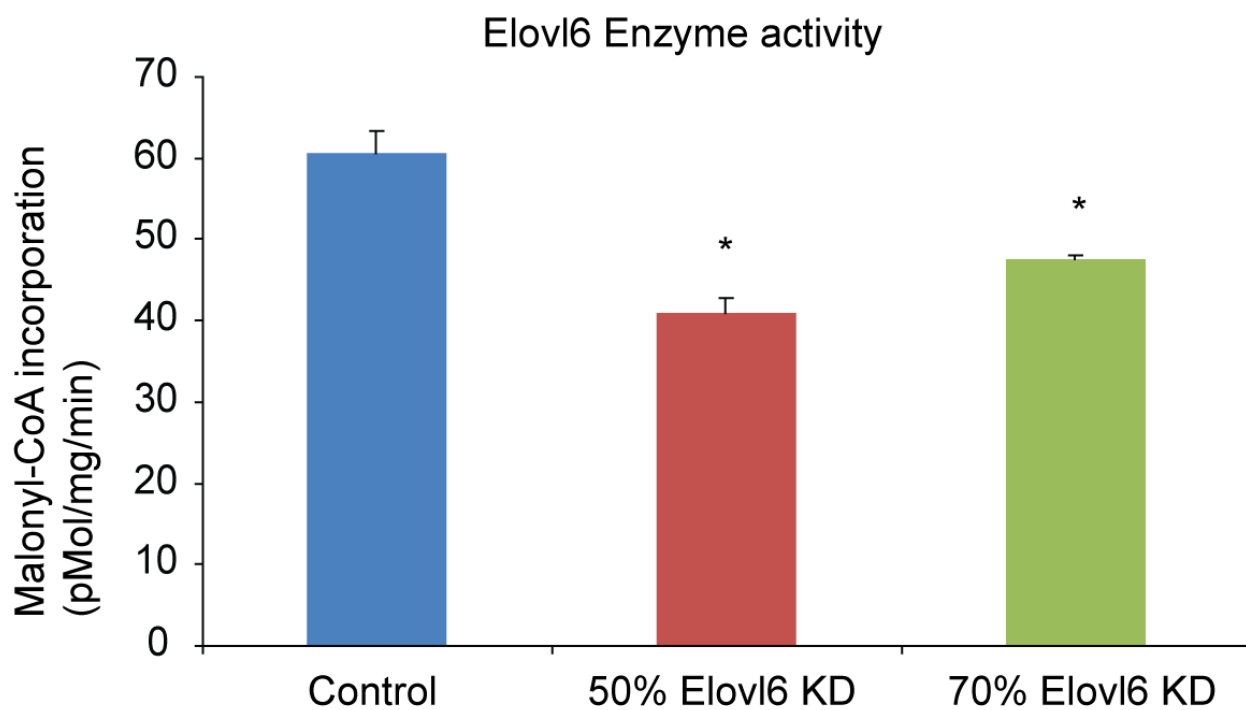

# B

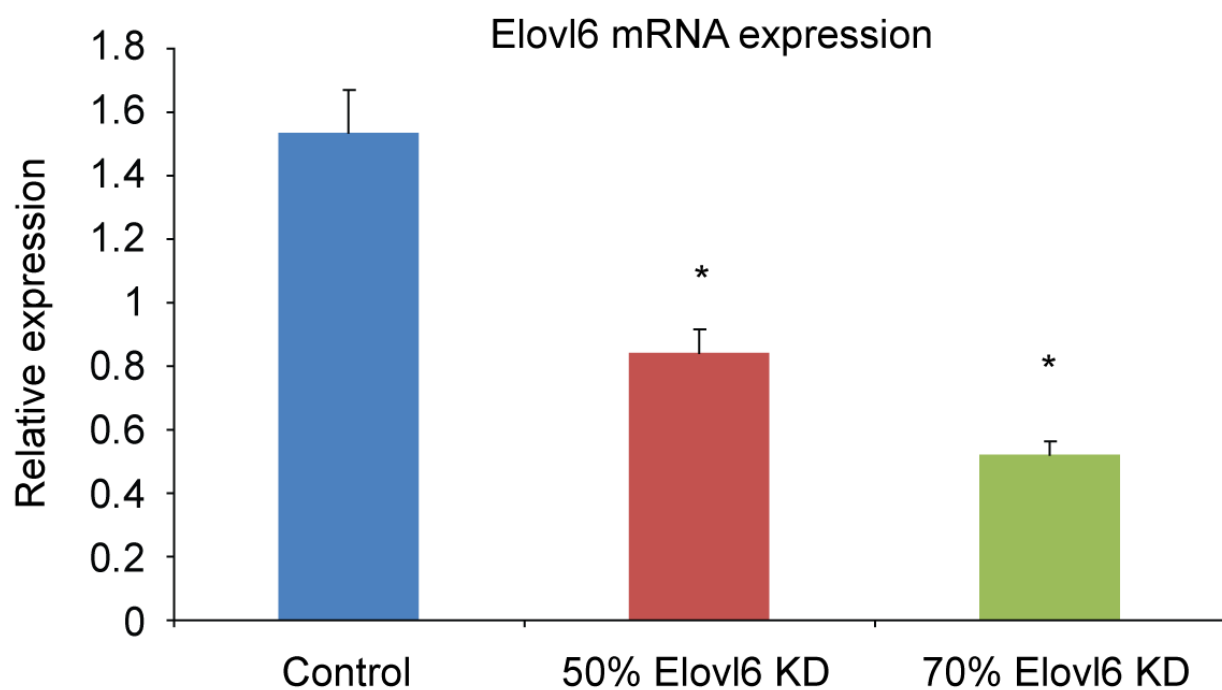

Supplement: Figure S7 — Elovl6 enzyme activity and mRNA expression in 3T3-L1 cell lines. (A) Elovl6 3T3-L1 knockdown cell lines have a functional reduction in C16:0–C18:0 fatty acid elongation ability. Elongation activity expressed as incorporation of radioactive malonyl-CoA into lipid fraction in picomoles per milligram of protein of isolated microsomes per minute. Palmitoyl-CoA was used as the substrate for elongation in the reaction. (B) Degree of knockdown of Elovl6 mRNA in 3T3-L1 cell lines. Expression in arbitrary units normalized to 18s housekeeping gene. *, p<0.05 versus control line, Student's t test followed by Bonferroni correction for multiple tests; n = 3 replicates per group. (0.04 MB PDF) [file pbio.1000623.s007.pdf]
