# Supplementary material for: Association of Lipidome Remodeling in the Adipocyte Membrane with Acquired Obesity in Humans
Source: PLoS Biol. 2011 Jun 7;9(6):e1000623. doi: 10.1371/journal.pbio.1000623 (PMC3110175; doi:10.1371/journal.pbio.1000623)
Supplement: Table S1 — Physical and biochemical characteristics of weight-discordant ( n = 13) and weight-concordant ( n = 9) monozygotic twin pairs. Data are median (interquartile range). aObese versus non-obese twins, paired Wilcoxon's test. b n = 9 discordant pairs. (0.04 MB DOC) [file pbio.1000623.s008.doc]

##

|  | **Discordant pairs** | | | **Concordant pairs** |
| --- | --- | --- | --- | --- |
|  | **Obese co-twins** | **Non-obese co-twins** | **p-value^a^** | **Both co-twins** |
| BMI (kg/m^2^) | 30.6 (29.2, 32.0) | 25.5 (24.8, 26.0) | 0.0015 | 26.1 (23.7, 29.4) |
| Percent body fat | 38.0 (32.6, 42.9) | 30.6 (29.2, 32.0) | 0.0015 | 30.6 (20.3, 37.3) |
| Subcutaneous fat (cm^3^) | 5113 (4376, 5821) | 2728 (2430, 3554) | 0.0015 | 3158 (1596, 4197) |
| Intra-abdominal fat (cm^3^) | 993 (827, 1065) | 516 (380, 587) | 0.0015 | 622 (361, 1251) |
| Liver fat (%) | 3.7 (2.0, 11.0) | 1.3 (1.0, 2.5) | 0.0016 | 1.0 (1.5, 9.0) |
| Fat cell size (μm) | 103.3 (95.5, 106.7) | 85.8 (82.5, 89.9) | 0.0022 | 91.3 (78.0, 97.0) |
| Fasting plasma glucose (mmol/l) | 5.5 (5.1, 5.9) | 5.1 (4.9, 5.4) | 0.033 | 5.6 (5.1, 5.7) |
| Fasting serum insulin (mU/l) | 9 (6, 11) | 4 (3, 6) | 0.016 | 6 (5, 10) |
| M value (mg·kg fat free mass^-1^·min^-1^) | 5.5 (4.5, 6.9) | 8.8 (7.6, 10.4) | 0.0047 | 7.2 (5.7, 8.2) |
| Serum adiponectin (μg/ml)^b^ | 7.6 (6.7, 12.6) | 11.0 (9.0, 14.9) | 0.028 | 9.8 (7.4, 11.9) |
| Serum leptin (ng/ml)^b^ | 24.1 (19.6, 37.4) | 15.1 (7.2, 18.8) | 0.0077 | 10.9 (5.6, 19.3) |
| Serum high sensitivity CRP (mg/l)^b^ | 2.2 (0.9, 3.2) | 0.9 (0.7, 1.0) | 0.0077 | 0.5 (0.2, 3.0) |
| Intake of polyunsaturated fatty acids (% of energy) | 3.2 (2.7, 4.1) | 4.5 (3.6, 5.7) | 0.050 | 4.0 (3.4, 4.4) |
