# Supplementary material for: Association of Lipidome Remodeling in the Adipocyte Membrane with Acquired Obesity in Humans
Source: PLoS Biol. 2011 Jun 7;9(6):e1000623. doi: 10.1371/journal.pbio.1000623 (PMC3110175; doi:10.1371/journal.pbio.1000623)
Supplement: Table S2 — Fatty acid composition in adipose tissue lipids of weight-discordant ( n = 13) and weight-concordant ( n = 9) monozygotic twin pairs. Data are median (interquartile range) (in molar precentage). aObese versus non-obese twins, paired t test. (0.04 MB DOC) [file pbio.1000623.s009.doc]

|  | **Discordant pairs** | | | **Concordant pairs** |
| --- | --- | --- | --- | --- |
| Fatty acid | **Obese co-twins** | **Non-obese co-twins** | **FDR q-value^a^** | **Both co-twins** |
| 12:0 | 0.14 (0.13, 0.21) | 0.23 (0.16, 0.25) | 0.016 | 0.23 (0.16, 0.27) |
| 14:0 | 2.55 (2.20, 3.20) | 2.72 (2.42, 3.09) | 0.163 | 3.01 (2.86, 3.31) |
| 14:1 | 0.36 (0.32, 0.40) | 0.28 (0.26, 0.30) | 0.007 | 0.31 (0.27, 0.33) |
| 15:0 | 0.29 (0.23, 0.33) | 0.27 (0.24, 0.30) | 0.065 | 0.29 (0.27, 0.32) |
| 16:0 | 22.14 (21.82, 24.26) | 22.56 (21.17, 23.19) | 0.058 | 22.46 (21.61, 22.91) |
| 16:1n-7 | 5.53 (5.37, 6.27) | 4.55 (3.97, 4.96) | <0.001 | 4.65 (4.21, 5.11) |
| 18:0 | 3.48 (3.28, 4.10) | 4.87 (4.20, 5.34) | <0.001 | 4.97 (4.53, 5.72) |
| 18:1 | 50.16 (49.03, 51.72) | 50.09 (49.66, 51.48) | 0.139 | 49.87 (49.12, 51.39) |
| 18:2n-6 | 9.92 (9.39, 10.05) | 10.40 (10.07, 11.24) | 0.006 | 9.80 (9.07, 10.10) |
| 18:3n-6 | 0.09 (0.09, 0.12) | 0.10 (0.08, 0.11) | 0.125 | 0.10 (0.09, 0.11) |
| 18:3n-3 | 1.13 (1.05, 1.24) | 1.22 (1.14, 1.36) | 0.017 | 1.17 (1.02, 1.36) |
| 18:4n-3 | 0.41 (0.39, 0.44) | 0.38 (0.34, 0.40) | 0.065 | 0.42 (0.37, 0.45) |
| 20:0 | 0.15 (0.13, 0.16) | 0.21 (0.20, 0.27) | <0.001 | 0.21 (0.18, 0.29) |
| 20:1 | 0.88 (0.87, 0.97) | 1.06 (0.99, 1.12) | <0.001 | 1.03 (0.95, 1.18) |
| 20:3n-6 | 0.19 (0.18, 0.24) | 0.17 (0.14, 0.18) | 0.004 | 0.15 (0.13, 0.17) |
| 20:4n-6 | 0.42 (0.38, 0.43) | 0.30 (0.27, 0.33) | <0.001 | 0.31 (0.24, 0.35) |
| 20:5n-3 | 0.11 (0.09, 0.13) | 0.08 (0.07, 0.09) | <0.001 | 0.08 (0.07, 0.10) |
| 22:5n-3 | 0.21 (0.18, 0.21) | 0.19 (0.16, 0.19) | 0.006 | 0.20 (0.17, 0.24) |
| 22:6n-3 | 0.15 (0.13, 0.21) | 0.14 (0.13, 0.22) | 0.171 | 0.15 (0.13, 0.25) |
