# Supplementary material for: Association of Lipidome Remodeling in the Adipocyte Membrane with Acquired Obesity in Humans
Source: PLoS Biol. 2011 Jun 7;9(6):e1000623. doi: 10.1371/journal.pbio.1000623 (PMC3110175; doi:10.1371/journal.pbio.1000623)
Supplement: Table S3 — Serum-free fatty acid composition in adipose tissue lipids of weight-discordant ( n = 13) and weight-concordant ( n = 9) monozygotic twin pairs. Data are median (interquartile range) (in µmol/l). aObese versus non-obese twins, paired t test. (0.04 MB DOC) [file pbio.1000623.s010.doc]

|  | **Discordant pairs** | |  | **Concordant pairs** |
| --- | --- | --- | --- | --- |
| Free fatty acid | **Obese co-twins** | **Non-obese co-twins** | **p-value^a^** | **Both co-twins** |
| 14:0 | 15.0 (13.2, 20.6) | 13.3 (10.2, 22.6) | 0.62 | 14.7 (10.0, 10.1) |
| 16:0 | 212.8 (200.4, 235.4) | 181.9 (134.5, 273.4) | 0.79 | 178.0 (162.2, 239.7) |
| 16:1 | 42.4 (31.6, 49.4) | 33.0 (25.7, 23.19) | 0.23 | 32.2 (22.1, 34.4) |
| 18:0 | 59.4 (53.5, 64.5) | 49.9 (41.4, 82.2) | 0.85 | 58.0 (53.8, 65.3) |
| 18:1n-9 | 271.0 (243.4, 314.8) | 236.2 (176.3, 383.0) | 0.96 | 244.1 (199.8, 318.2) |
| 18:1n-7 | 30.2 (25.0, 37.3) | 22.0 (20.8, 45.5) | 0.88 | 24.9 (20.4, 41.0) |
| 18:2n-6 | 140.9 (126.0, 174.7) | 151.9 (115.2, 304.4) | 0.96 | 131.3 (98.7, 199.6) |
| 18:3n-6 | 1.9 (1.5, 2.5) | 2.2 (1.3, 3.3) | 0.65 | 1.7 (1.0, 2.1) |
| 18:3n-3 | 13.1 (11.0, 14.5) | 10.4 (8.3, 17.5) | 0.93 | 10.9 (8.1, 13.3) |
| 20:3n-6 | 3.3 (3.0, 4.0) | 2.7 (2.4, 3.8) | 0.25 | 2.4 (2.1, 2.9) |
| 20:4n-6 | 21.5 (19.4, 24.3) | 20.4 (15.3, 33.2) | 0.75 | 17.5 (15.2, 25.2) |
| 20:5n-3 | 4.4 (4.0, 4.7) | 4.2 (3.4, 5.4) | 0.65 | 3.7 (2.7, 4.9) |
| 22:5n-3 | 1.9 (1.7, 2.0) | 1.9 (1.6, 2.0) | 0.45 | 1.8 (1.4, 2.1) |
| 22:6n-3 | 7.8 (7.1, 10.4) | 8.6 (6.8, 11.7) | 0.94 | 8.0 (7.2, 9.2) |
