# Supplementary material for: Association of Lipidome Remodeling in the Adipocyte Membrane with Acquired Obesity in Humans
Source: PLoS Biol. 2011 Jun 7;9(6):e1000623. doi: 10.1371/journal.pbio.1000623 (PMC3110175; doi:10.1371/journal.pbio.1000623)
Supplement: Table S4 — Selected variables included in the dependency network analysis. (0.10 MB DOC) [file pbio.1000623.s011.doc]

| **Variable name** | **p-value**  **(Pairwise**  ***t*- test)** | **Fold**  **(log2)** | **Description** |  |  |
| --- | --- | --- | --- | --- | --- |
| **Clinical variables** | | | | | |
| BMI | 0.00000024 | 0.21 | Body mass index |  |  |
| SCfat | 0.00000284 | 0.71 | Subcutaneous fat |  |  |
| FCsize | 0.00000339 | 0.20 | Fat cell size |  |  |
| Fat% | 0.00001489 | 0.37 | Fat percentage |  |  |
| IAfat | 0.00003490 | 1.20 | Intra-abdominal fat |  |  |
| Mvalue | 0.00111025 | -0.35 | M-value |  |  |
| Insulin | 0.00457518 | 1.01 | Insulin |  |  |
| Lfat | 0.00611285 | 2.74 | Liver fat |  |  |
| PUFA% | 0.04859691 | -0.16 | PUFA % dietary intake |  |  |
| C16:1n-7 | 0.22536008 | 0.48 | Serum C16:1n-7 free fatty acid |  |  |
| **Adipose tissue lipids** | | | | | |
| PE(P-16:0/20:4) | 0.00005517 | 0.32 | Ethanolamine plasmalogen  PE(P-16:0/20:4) |  |  |
| PC(34:2) | 0.00259720 | -0.15 | Phosphatidylcholine PC(34:2) |  |  |
| Cer(d18:1/24:0) | 0.10126967 | 0.35 | Ceramide Cer(d18:1/24:0) |  |  |
| FChol | 0.23871729 | 0.09 | Free cholesterol |  |  |
| lysoPC(18:0) | 0.85628725 | 0.10 | Lysophosphatidylcholine  lysoPC(18:0) |  |  |
| C20:4n6 | 0.00000417 | 0.33 | Arachidonic acid (esterified) |  |  |
| C16:1n-7 | 0.00004483 | 0.28 | Palmitoleic acid (esterified) |  |  |
| 16:1/16:0 | 0.00059869 | 0.25 | Ratio of palmitoleic to  palmitic acids (esterfied) |  |  |
| 22:5/20:5 | 0.00101263 | -0.14 | Ratio of C22:5 and C20:5  fatty acids (esterified) |  |  |
| 20:3/18:3n7 | 0.02130082 | 0.21 | Ratio of C20:3 and C18:3n7  fatty acids (esterified) |  |  |
| **Adipose tissue gene lists** | | | | | |
| InflamRes | 0.00122198 | 0.82 | Inflammatory response pathway |  |  |
| BCAAcat | 0.01036096 | -0.73 | Branched chain amino acid  catabolism pathway |  |  |
| **Adipose tissue genes** | | | | **Entrez**  **ID** | **Probe ID** |
| ACACB | 0.03869739 | -0.12 | acetyl-Coenzyme A carboxylase  beta | 32 | _43427_atacacb |
| ACADM | 0.01295672 | -0.17 | acyl-Coenzyme A dehydrogenase,  C-4 to C-12 straight chain | 34 | _202502_atacadm |
| ADIPOQ | 0.15283243 | -0.05 | adiponectin, C1Q and collagen  domain containing | 9370 | _207175_ataadipon |
| ADIPOR1 | 0.79878942 | 0.04 | adiponectin receptor 1 | 51094 | _217748_atadipor1 |
| ADIPOR2 | 0.39176918 | 0.07 | adiponectin receptor 2 | 79602 | _201346_atadipor2 |
| AGPAT9 | 0.01269124 | -0.56 | 1-acylglycerol-3-phosphate O-  acyltransferase 9 | 84803 | _224480_s_atagpat9 |
| AGPS | 0.36236553 | 0.21 | alkylglycerone phosphate synthase | 8540 | _205401_atagps |
| CCND1 | 0.00905852 | 0.87 | cyclin D1 | 595 | _208711_s_atccnd1 |
| CD36 | 0.46850821 | -0.02 | CD36 molecule (thrombospondin  receptor) | 948 | _215835_atscarb1 |
| CEPT | 0.88574613 | 0.22 | cholesteryl ester transfer protein | 1071 | _206210_s_atcetp |
| CPT1B | 0.97631098 | 0.14 | carnitine palmitoyltransferase 1B  (muscle) | 1375 | _210069_atcpt1b |
| ELOVL3 | 0.16071989 | 0.32 | elongation of very long chain fatty  acids | 83401 | _234513_atelovl3 |
| ELOVL4 | 0.96869249 | 0.04 | elongation of very long chain fatty  acids (FEN1/Elo2, SUR4/Elo3,  yeast)-like 4 | 6785 | _219532_atelovl4 |
| ELOVL5 | 0.63583783 | 0.04 | ELOVL family member 5, elongation  of long chain fatty acids | 60481 | _208788_atelovl5 |
| ELOVL6 | 0.62231242 | 1.03 | ELOVL family member 6, elongation  of long chain fatty acids | 79071 | _227491_atelovl6 |
| FABP4 | 0.50046485 | -0.01 | fatty acid binding protein 4,  adipocyte | 2167 | _235978_atfabp4 |
| FASN | 0.49673183 | 0.35 | fatty acid synthase | 2194 | _212218_s_atfasn |
| FRP1 | 0.00356636 | 0.53 | secreted frizzled-related protein 1 | 6422 | _209902_atfrp1 |
| GLUT4 | 0.12608174 | -0.05 | solute carrier family 2 (facilitated  glucose transporter), member 4 | 6517 | _206603_atglut4 |
| GNPAT | 0.16539421 | -0.05 | glyceronephosphate O-  acyltransferase | 8443 | _201956_s_atgnpat |
| INSIG1 | 0.39378120 | 0.35 | insulin induced gene 1 | 3638 | _201627_s_atinsig1 |
| IRS2 | 0.10560220 | -0.08 | insulin receptor substrate 2 | 8660 | _209184_s_atirs2 |
| LPL | 0.38396189 | -0.03 | lipoprotein lipase | 4023 | _203549_s_atlpl |
| PCCB | 0.01398639 | -0.22 | propionyl Coenzyme A carboxylase,  beta polypeptide | 5096 | _212694_s_atpccb |
| PEMT | 0.00093906 | 1.21 | phosphatidylethanolamine N-  methyltransferase | 10400 | _207621_s_atpemt |
| PLA2G6 | 0.90719078 | 0.13 | phospholipase A2, group VI  (cytosolic, calcium-independent) | 8398 | _210647_x_atpla2g6 |
| PMVK | 0.00008246 | 0.31 | phosphomevalonate kinase | 10654 | _203515_s_atpmvk |
| PPARG | 0.03651163 | -0.18 | peroxisome proliferator-activated  receptor gamma | 5468 | _208510_s_atpparg |
| SCD1 | 0.20869350 | -0.08 | stearoyl-CoA desaturase (delta-9-  desaturase) | 6319 | _200831_s_atscd |
| SREBP-1c | 0.97665162 | 0.15 | sterol regulatory element binding  transcription factor 1 | 6720 | _201247_atsrebf1 |
| VLDLR | 0.13695974 | 1.04 | very low density lipoprotein  receptor | 7436 | _1558212_atvldlr |
