# Supplementary material for: Association of Lipidome Remodeling in the Adipocyte Membrane with Acquired Obesity in Humans
Source: PLoS Biol. 2011 Jun 7;9(6):e1000623. doi: 10.1371/journal.pbio.1000623 (PMC3110175; doi:10.1371/journal.pbio.1000623)
Supplement: Table S5 — Top-ranking phospholipids (PC and PE class) differentiating mature adipocytes in Elovl6 70% knockdown cell line as compared to controls. (0.04 MB DOC) [file pbio.1000623.s012.doc]

## Table S5. Top ranking phospholipids (PC and PE class) differentiating mature adipocytes in Elovl6 70% knock-down (KD) cell line as compared to controls.

| **Lipid name** | **Log2 old change**  **(70% Elovl6 KD vs Control in mature adipocytes at 8 days)** | **p-value**  **(ANOVA group**  **effect)** |
| --- | --- | --- |
| PE(O-40:6) | -1.26 | 0.025 |
| PE(38:0) | -0.92 | 0.010 |
| PC(O-32:2) | -0.61 | 0.016 |
| PC(33:0) | -0.52 | 0.030 |
| PC(40:7) | -0.50 | 0.041 |
| PC(O-40:4) | -0.38 | 0.034 |
| PE(O-38:7) | -0.21 | 0.043 |
| PC(38:4) | -0.18 | 0.026 |
| PC(36:3) | 0.07 | 0.022 |
| PC(38:2) | 0.08 | 0.034 |
| PC(40:4) | 0.09 | 0.012 |
| PC(34:2) | 0.19 | 0.010 |
| PC(O-34:1) | 0.19 | 0.011 |
| PC(36:6) | 0.21 | 0.031 |
| PC(O-38:3) | 0.26 | 0.015 |
| PC(40:3) | 0.29 | 0.007 |
| PE(36:4) | 0.29 | 0.027 |
| PC(34:3) | 0.34 | 0.029 |
