# Supplementary material for: Association of Lipidome Remodeling in the Adipocyte Membrane with Acquired Obesity in Humans
Source: PLoS Biol. 2011 Jun 7;9(6):e1000623. doi: 10.1371/journal.pbio.1000623 (PMC3110175; doi:10.1371/journal.pbio.1000623)
Supplement: Text S1 — Experimental methods in the twin study. (0.04 MB DOC) [file pbio.1000623.s013.doc]

**Text S1: Experimental methods in the twin study**

All subjects were studied starting at 8 a.m after an overnight fast. Venous blood samples were obtained and plasma with ethylenediaminetetraacetate (EDTA) and serum were separated by centrifugation and stored at -80°C until the analyses of insulin, adiponectin, leptin, and high sensitivity CRP. Whole body insulin sensitivity (the M-value) was measured using the euglycemic hyperinsulinemic clamp technique as described . Body composition was measured by dual-energy x-ray absorptiometry (DEXA), subcutaneous and intra-abdominal fat by magnetic resonance imaging (MRI) of 16 transaxial scans reaching from 8 cm above to 8 cm below the fourth and fifth lumbar interspace and liver fat content by proton spectroscopy . Fat cell size was determined from fresh subcutaneous abdominal adipose tissue samples treated with collagenase. Gene expression analysis of adipose tissue in weight discordant twin pairs was previously reported . In short, RNA was prepared from frozen fat and used for the global gene expression analyses in Affymetrix U133 Plus 2.0 chips. Affymetrix gene expression chip data was analyzed using MAS-5 according to manufacturer’s recommendations, and preprocessed with the GC-RMA algorithm.

**REFERENCES**

1. DeFronzo RA, Tobin JD, Andres R (1979) Glucose clamp technique: a method for quantifying insulin secretion and resistance. Am J Physiol 237: E214-E223.

2. Pietiläinen KH, Rissanen A, Kaprio J, Mäkimattila S, Häkkinen AM, et al. (2005) Acquired obesity is associated with increased liver fat, intra-abdominal fat, and insulin resistance in young adult monozygotic twins. Am J Physiol Endocrinol Metab 288: E768-E774.

3. Pietiläinen KH, Naukkarinen J, Rissanen A, Saharinen J, Ellonen P, et al. (2008) Global transcript profiles of fat in monozygotic twins discordant for BMI: pathways behind acquired obesity. PLoS Med 5: e51.
