# Supplementary material for: Association of Lipidome Remodeling in the Adipocyte Membrane with Acquired Obesity in Humans
Source: PLoS Biol. 2011 Jun 7;9(6):e1000623. doi: 10.1371/journal.pbio.1000623 (PMC3110175; doi:10.1371/journal.pbio.1000623)
Supplement: Text S2 — Characerization of Elovl6 knockdown in vitro. (0.04 MB DOC) [file pbio.1000623.s014.doc]

## Text S2: Characerization of Elovl6 knock-down *in vitro*

### Preparations of Microsomes and the Fatty Acid Elongation Assay

25x10^7 3t3-L1 cells were homogenised using a Cell Cracker (Isobiotech, Hiedelberg, Germany). Following a 30-min stepwise centrifugation (10 min at 700 × g, 8,000 × g, and 17,000 × g) at 4°C, the supernatant was carefully transferred to fresh tubes, and microsomes were sedimented at 105,000 × g for 45 min. The pellet was resuspended in 20 mm Tris-HCl (pH 7.4) containing 0.4 m KCl and centrifuged at 105,000 × g for 45 min. The final microsomal pellet was resuspended in 200 μl of 0.1 m Tris-HCl (pH 7.4), and the protein concentration was determined.

Total fatty acid elongation activity was measured essentially according to Suneja et al. . The assay mixtures (0.1 ml total, including a 50 μg protein addition) contained 0.1 m Tris-HCl (pH 7.4); 50 μm palmitoyl-CoA (C16:0); 100 um Fatty acid free BSA (Sigma); 1 mm NADPH; and 50 μm malonyl CoA (containing 0.02 μCi of [2-14C]malonyl-CoA). The reaction was carried out for 20 min at 37 °C and terminated by the addition of 0.1 ml of 5M aqueos KOH containing 10% methanol and saponified at 65 °C for 45 min. The samples were cooled and acidified with 0.1 ml of cold 5 m HCl, and then 0.1 ml of EtOH was added. Free fatty acids were extracted from the mixture twice with 0.5ml of n-hexane containing 2% glacial acetic acid. The solvent extracts were added to 9 ml of organic compatible scintillation mixture and activity was analyzed in a Beckman liquid scintillation system 3801.

### RNA extraction and real-time PCR

RNA was extracted from 3t3-L1 adipocytes using the RNAeasy kit (Qiagen) according to the manufacturer’s instructions. RNA concentration was determined using nanodrop spectrophotometer (Thermo Fisher scientific, Delaware USA). 500 ng of RNA was used to generate cDNA according to the manufacturer’s protocol (Reverse Transcriptase System, Promega). Real-Time PCR was performed using TaqMan or Sybr green (Applied Biosystems) on an ABi 7900 Taqman machine using default thermal cycling conditions. For Sybr Green reactions a melting curve analysis was carried out to confirm the presence of a single product.

All primers were designed on Primer Express v3.0.

Primers for Sybr Green

Elovl6 Forward: ggaggagagcccctgagcta

Elovl6 Reverse: tgatcttcggagtcgctacgt

Primers for TaqMan

18s Forward: CGGCTACCACATCCAAGGAA
18s Reverse: GCTGGAATTACCGCGGCT

18s Probe: GAG GGC AAG TCT GGT GCC AG

**REFERENCES**

1. Suneja SK, Nagi MN, Cook L, Cinti DL (1991) Decreased long-chain fatty acyl CoA elongation activity in quaking and jimpy mouse brain: deficiency in one enzyme or multiple enzyme activities? J Neurochem 57: 140-146.
