# Supplementary material for: Association of Lipidome Remodeling in the Adipocyte Membrane with Acquired Obesity in Humans
Source: PLoS Biol. 2011 Jun 7;9(6):e1000623. doi: 10.1371/journal.pbio.1000623 (PMC3110175; doi:10.1371/journal.pbio.1000623)
Supplement: Text S3 — Methods for lipidomic analysis. (0.05 MB DOC) [file pbio.1000623.s015.doc]

**Text S3: Lipidomic analysis**

### Lipidomic analysis of adipose tissue biopsies

Frozen fat tissue samples (5 mg) were mixed with an internal standard mixture 1 (IS1) containing lysoPC(17:0), Cer(d18:1/17:0), PC(17:0/17:0), PE(17:0/17:0) and TG(17:0/17:0/17:0) at concentration level of 0.5-1 µg/sample and 200 µl chloroform:methanol (2:1). The tissues were homogenized with grinding balls in a Retsch mixer mill MM400 homogenizer at 25 Hz for 2 min and 50 µl of 0.9% NaCl was added. The samples were vortexed for 2 min and after 30 min standing centrifuged at 10 000 rpm for 3 min. The labelled lipid standard mixture (IS2) containing PC (16:1/0:0-D_3_), PC(16:1/16:1-D_6_) and TG(16:0/16:0/16:0-^13^C3) was added into the separated lipid extracts (1 µg/sample) before UPLC-MS analysis.

Lipid extracts were analyzed on a Waters Q-Tof Premier mass spectrometer combined with an Acquity Ultra Performance LC^TM^ (UPLC). The column (at 50°C) was an Acquity UPLC^TM^ BEH C18 1 × 50 mm with 1.7 μm particles. The solvent system included A. ultrapure water (1% 1 M NH_4_Ac, 0.1% HCOOH) and B. LC/MS grade acetonitrile/isopropanol (5:2, 1% 1M NH_4_Ac, 0.1% HCOOH). The gradient started from 65% A / 35% B, reached 100% B in 6 min and remained there for the next 7 min. There was a 5 min re-equilibration step before the next run. The flow rate was 0.200 ml/min and the injected amount was 1.0 μl (Acquity Sample Organizer). Reserpine was used as the lock spray reference compound. The lipid profiling was carried out using ESI+ mode and the data was collected at mass range of m/z 300-1200 with a scan duration of 0.2 sec. The data was processed using MZmine software (version 6.0) and the lipid identification was based on an internal spectral library.

### Lipidomic analysis of 3T3-L1 adipocytes

The cell suspension samples were centrifuged and the supernatants were removed. The cell residues were homogenized with 50 µl of BSA-buffer by using a Retsch homogenizator (2 grinding balls Ø 3mm, 20 Hz, 2 min). 5 µl of the homogenate was separated for protein determination and the rest of the homogenate was spiked with an internal lipid standard mixture IS1 (10 lipids at concentration level of 0.4-0.6 µg/sample) and the lipids were extracted with chloroform:methanol (100 µl, 2:1) by further homogenizing the sample for 2 min. After 30 min the samples were centrifuged at 10 000 rpm for 3 min and the lower layer was separated (60 µl) and spiked with a labeled lipid mixture (IS2, 0.1-0.2 µg/sample).

Lipid extracts were analyzed on a Waters Q-Tof Premier mass spectrometer combined with an Acquity Ultra Performance LC^TM^ (UPLC). The column (at 50 °C) was an Acquity UPLC^TM^ BEH C18 2.1 × 100 mm with 1.7 μm particles. The solvent system included A. ultrapure water (1% 1 M NH_4_Ac, 0.1% HCOOH) and B. LC/MS grade acetonitrile/isopropanol (1:1, 1% 1M NH_4_Ac, 0.1% HCOOH). The gradient started from 65% A / 35% B, reached 80% B in 2 min, 100% B in 7 min and remained there for 7 min. The flow rate was 0.400 ml/min and the injected amount was 2.0 μl (Acquity Sample Organizer, at 10 °C). Reserpine was used as the lock spray reference compound. The lipid profiling was carried out using ESI+ mode and the data was collected at mass range of m/z 300-1200 with scan duration of 0.2 sec. The data was processed by using MZmine 2 software and the lipid identification was based on an internal spectral library.

### Lipid identification

The UPLC/MS system consisted of an Acquity Ultra Performance LC^TM^ (UPLC) combined with a LTQ-Orbitrap mass spectrometer (Thermo Scientific). A sample organizer was used for the automatic sampling. The temperature of the sample organizer was 10°C. The column was an Acquity UPLC® BEH C18 1.7 µm 2.1 × 100 mm column from Waters Inc. (Ireland). The column was kept at 50°C. The analysis was performed in gradient mode, using two solvents as follows: A. 1% 1M NH4Ac, 0.1% HCOOH in water and B. AcCN / 2-Propanol (1:1; 1% 1M NH4Ac, 0.1% HCOOH). The gradient program started at 65% A / 35% B, set to reach 80% B in 2 min, 100% B in 7 min and remained there for 7 min. The total running time including a 4 min re-equilibration step was 18 min. The flow rate was 0.400 ml/min and the injected amount of lipid extract was 2 μl.

The MS was equipped with electrospray ionization source and it was applied in either positive or negative ion mode. The data was collected in continuum mode using extended dynamic range at mass range of m/z 300-1200. The instrument is calibrated externally according to the instructions of the manufacturer. MS^2^ and MS^3^ are acquired using either low resolution (LTQ) or high resolution up to target mass resolution R = 60 000 at m/z 400. The normalized collision energies of 30% and 35% are applied in MS^2^ andMS^3^ experiments, respectively.

### Fatty acid analysis in adipose tissue by Gas Chromatography –flame ionization detector (GC-FID)

Fat tissues samples (5 mg) were spiked with 80 µg of internal standards (TG(17:0/17:0/17:0); FFA 17:0) and homogenized in capped microtubes (Sarstedt) at -20˚C with chloroform: methanol (2:1; 800 μl) by using zirconium oxide balls (one 5 mm and two 3 mm) in a Mixer Mill 301 (25 Hz for 5 min). 200 μl NaCl (0.9 %) was added by vortexing and after an extraction time of 1 hr, the lower layer was separated by centrifuging at 10 000 rpm for 5 min. 100 μl aliquots from the extracts were evaporated into dryness and used for fatty acid analyses.

The evaporation residues from lipid extractions were dissolved into petroleum ether (b.p. 40-60 °C; 700 µl). Fatty acids were transmethylated with sodium methoxide (NaOMe; 0.5 M; 250 µl) in dry methanol by boiling at 45 °C for 5 min. The mixture was acidified with 15% sodium hydrogen sulphate (NaHSO4; 500 µl) and the petroleum ether phase containing the fatty acid methyl esters (FAME) as well as free fatty acids (FFA) was collected. After centrifugation (10 000 rpm, 5 min) the petroleum ether layer was separated into a GC vial and evaporated, the residue was dissolved into hexane (100 µl) and 2 µl aliquot was used for GC analysis.

Agilent 5890 series II GC was equipped with a 25 m FFAP column (0.32 mm ID) and helium was used as carrier gas. The temperatures of the GC injector (injection volume 1 μl with split ratio 1:23) and detector (FID) were 260°C and 300°C, respectively, and the oven was programmed from 70°C to 240 °C at 7°C/min.

### GC-MS SIM Method for free cholesterol determination in adipose tissue

Frozen (-80°C) adipose fat tissue samples (5 mg) were spiked with 20 µg of labeled cholesterol and homogenized in capped microtubes at -20˚C with chloroform: methanol (2:1; 600 µl) by using zirconium oxide balls (one 5 mm and two 3 mm) in a Mixer Mill 301 (25 Hz for 5 min). Samples were mixed with 150 µl NaCl (0.9 %) and after 30 min extraction time the lower layer was separated by centrifuging at 10 000 rpm for 5 min. The samples were evaporated and the residues were derivatized with MSTFA (50 µl) by incubating at 45 °C for 1 h.

The samples were run on an Agilent 6890 gas chromatograph (GC) combined with Agilent 5973 mass selective detector (MSD). The injector (injection volume 1μl with split ratio 60:1) and MSD interface temperatures were 280 °C, and the oven temperature programme was from 100 °C to 280 °C at a rate of 20°C/min. The analyses were performed on an Agilent Ultra-2 (5% phenyl methyl siloxane) capillary column (25 m, ID 200 µm, film thickness 0.33 µm; Agilent 19091B-102). Cholesterol and labeled cholesterol were detected by monitoring m/z 374 and m/z 368.

### Serum free fatty acid analysis by GC-MS

Serum samples (50 µl) were diluted with 100 µl of 0.9% sodium chloride and spiked with heptadecanoic acid (FFA 17:0; 2 µg) and extracted with chloroform:methanol (2:1 v/v; 400 µl) by vortexing for 1 min. After 30 min standing, the samples were centrifuged at 10 000 rpm for 5 min and the lower layer was separated and evaporated into dryness under nitrogen flow. The evaporation residues from lipid extractions were dissolved into petroleum ether (b.p. 40-60°C; 700 µl). Fatty acids were transmethylated with sodium methoxide (NaOMe; 0.5 M; 250 µl) in dry methanol by boiling at 45°C for 5 min. The mixture was acidified with 15% sodium hydrogen sulphate (NaHSO4; 500 µl) and the petroleum ether phase containing the free fatty acids (FFA) was collected. . After centrifugation (10 000 rpm, 5 min) the petroleum ether layer was separated into a GC vial and evaporated. The dried residue was derivatized with BSTFA (40 µl) and TMCS (10 µl) by incubating at 80°C for 2 hr. The samples (1 µl aliquots) were analyzed on an Agilent 6890 gas chromatograph (GC) combined with Agilent 5973 mass selective detector (MSD). A splitless injection method was used and the injector and MSD interface temperatures were 260°C and 280°C, respectively. FFAs were analyzed on an Agilent Ultra-2 (5% phenyl methyl siloxane) capillary column (25 m, ID 200 µm, film thickness 0.33 µm; Agilent 19091B-102) by using helium as carrier gas. FFAs were monitored at m/z 285 (14:0), 311 (16:1), 313 (16:0), 335 (18:3n-6), 337 (18:2n-6), 339 (18:1n-9), 335 (18:3n-3), 339 (18:1n-7), 341 (18:0), 361 (20:4n-6), 359 (20:5n-3), 363 (20:3n-6), 387 (22:5n-6), 385 (22:6n-3) and m/z 387 (22:5n-3).

**REFERENCES**

1. Katajamaa M, Miettinen J, Oresic M (2006) MZmine: toolbox for processing and visualization of mass spectrometry based molecular profile data. Bioinformatics 22: 634-636.

2. Katajamaa M, Oresic M (2005) Processing methods for differential analysis of LC/MS profile data. BMC Bioinformatics 6: 179.

3. Pluskal T, Castillo S, Villar-Briones A, Oresic M (2010) MZmine 2: Modular framework for processing, visualizing, and analyzing mass spectrometry-based molecular profile data. BMC Bioinformatics 11: 395.
