# Supplementary material for: Association of Lipidome Remodeling in the Adipocyte Membrane with Acquired Obesity in Humans
Source: PLoS Biol. 2011 Jun 7;9(6):e1000623. doi: 10.1371/journal.pbio.1000623 (PMC3110175; doi:10.1371/journal.pbio.1000623)
Supplement: Text S4 — Computational and statistical methods. (0.06 MB DOC) [file pbio.1000623.s016.doc]

## Text S4: Computational and statistical methods

### Cluster analysis

The data was scaled into zero mean and unit variance to obtain lipid profiles comparable to each other. Model-based clustering was then applied on the scaled data for grouping similarly expressed lipids across the sampling conditions. The analyses were performed using MCLUST which is among the most powerful Bayesian methods for high dimensional multivariate analyses, which is implemented in R statistical language (http://www.r-project.org/) as package “mclust”. In MCLUST the observed data is viewed as a mixture of several clusters and each cluster comes from a unique probability density function. The number of clusters in the mixture, together with the cluster-specific parameters that constrain the probability distributions, will define a model which can then be compared to others. The clustering process therefore selects the optimal model and determines the data partition accordingly.

In practice we considered the number of clusters ranging from 2 to 15, and included the complete model families that are available in MCLUST. Model comparison was carried out *via* BIC (Bayesian information criterion) which is an approximation of the marginal likelihood. Therefore the best model is the one which gives the largest marginal likelihood of data, i.e. the highest BIC value.

### Partial correlation network analysis

We combined N=60 variables of different domains measured for the n=26 weight-discordant co-twins (*i.e*., 13 twin pairs), including 9 clinical variables, 6 fatty acids, 5 lipids, 2 pathway profiles and 38 transcripts. The proportion of missing data is 0.4%. If a variable for both twins in a pair is missing, it was imputed by sampling a normal distribution with the mean and variance estimated from the non-missing twin pairs. If either twin was missing, it was imputed with the same observation for the co-twin. Such an imputation method might bring potential bias, but its effect should be negligible due to the small missing percentage .

Direct estimation of partial correlation is difficult for such data, as the higher number of variables relative to the sample size prevents reliable estimation of the joint probability distribution of the conditional set . One solution is to decrease the size of conditional set down to a lower order q < (n-2) to approximate the entire full-order partial correlation . Structure learning of the Gaussian graphs therefore corresponds to a statistical test such as t-test for the hypothesis that a given q-order partial correlation is zero. If all of such hypotheses of zero q-order partial correlations are rejected, then the two variables are joined by an edge. In practice, we tested the hypothesis by default with 4 equidistant q values along the [1, 24] interval, namely q = 1, 7, 12 and 18. For each of the q values, the test was repeated for each pair of variables by sampling 500 elements randomly selected from the subsets of the data which contain q variables. A missing edge is identified if the proportion of such tests where the null-hypothesis is not rejected, *e.g.* the average non-rejection rate of the hypothesis, is above a certain threshold β. A small average non-rejection rate therefore implies a strong evidence of dependence. For generation of the network in Figure 4 we used β=0.55. The resulting graph thus can be obtained by removing all the missing edges from the complete graph.

**Lipid bilayer simulations**

To parameterize the lipid molecules, we used the all-atom OPLS (Optimized Parameters for Liquid Simulations) force-field . Since the force field was not originally developed for lipids we re-parameterized all torsional angles in the glycerol region (details will be published elsewhere). For water, we employed the TIP3P model, which is compatible with the OPLS parameterization . This combination of force field was successfully used in our previous studies .

Periodic boundary conditions with the usual minimum image convention were used in all three directions. The LINCS algorithm was used to preserve the hydrogen covalent bond lengths. Time step was set to 2 fs and the simulations were carried out at constant pressure (1 bar) and temperature (310 K), the latter choice implying that the systems are in the fluid phase. The temperature and pressure were controlled using the Nosé-Hoover and the Parrinello-Rahman methods, respectively. For pressure we used semi-isotropic control. The Lennard-Jones interactions were cut off at 1.0 nm and for the electrostatic interactions we employed the particle-mesh Ewald method . The list of non-bonded pairs was updated every 10th time step.

To describe lipid bilayer properties, following parameters were calculated: surface area, bilayer thickness, and order parameter S_mol_. The order parameter essentially provides the same information as the ^2^H NMR (or ^1^H NMR) order parameters determined through selective deuteration. The surface area (per lipid) was calculated by dividing the total area of the bilayer by the number of lipids in a single leaflet. In the cases of mixed bilayers, this quantity does not provide information about the difference between the individual components, but it allows comparison between the mixed systems. For bilayer thickness, the P-P (phosphorous-phosphorous) distance was measured between the average positions of the phosphate atoms in opposite leaflets. S_mol_ parameter was calculated according to the following formula:

where *θ_n_* is an instantaneous angle between the *n*th segmental vector, i.e., (C_n-1_, C_n+1_) vector linking *n –* 1 and *n +* 1 carbon atoms in the acyl (alkyl) chain and the bilayer normal; < > denotes both the ensemble and the time average. The standard errors of the mean were calculated by using the block analysis method as described in .

**REFERENCES**

1. Fraley C, Raftery AE (2007) Model-based methods of classification: Using the mclust software in chemometrics. J Stat Soft 18: 1-13.

2. Cui J, Sheffield L (2003) Bivariate variance-component analysis, with application to systolic blood pressure and total cholesterol levels in the Framingham Heart Study. BMC Genet 4: S81.

3. Whittaker J (1990) Graphical models in applied multivariate statistics. Chichester: Wiley.

4. Edwards DE (2000) Introduction to graphical modeling. New York: Springer Verlag.

5. Jorgensen WL, Maxwell DS, Tirado-Rives J (1996) Development and Testing of the OPLS All-Atom Force Field on Conformational Energetics and Properties of Organic Liquids. Journal of the American Chemical Society 118: 11225-11236.

6. Rizzo RC, Jorgensen WL (1999) OPLS All-Atom Model for Amines: Resolution of the Amine Hydration Problem. Journal of the American Chemical Society 121: 4827-4836.

7. Price MLP, Ostrovsky D, Jorgensen WL (2001) Gas-phase and liquid-state properties of esters, nitriles, and nitro compounds with the OPLS-AA force field. Journal of Computational Chemistry 22: 1340-1352.

8. Kaminski GA, Friesner RA, Tirado-Rives J, Jorgensen WL (2001) Evaluation and Reparametrization of the OPLS-AA Force Field for Proteins via Comparison with Accurate Quantum Chemical Calculations on Peptides. The Journal of Physical Chemistry B 105: 6474-6487.

9. Jorgensen WL, Chandrasekhar J, Madura JD, Impey RW, Klein ML (1983) Comparison of simple potential functions for simulating liquid water. The Journal of Chemical Physics 79: 926-935.

10. Rog T, Vattulainen I, Bunker A, Karttunen M (2007) Glycolipid Membranes through Atomistic Simulations: Effect of Glucose and Galactose Head Groups on Lipid Bilayer Properties. The Journal of Physical Chemistry B 111: 10146-10154.

11. Rog T, Martinez-Seara H, Munck N, Oresic M, Karttunen M, et al. (2009) Role of cardiolipins in the inner mitochondrial membrane: insight gained through atom-scale simulations. The Journal of Physical Chemistry B 113: 3413-3422.

12. Hess B, Bekker H, Berendsen HJC, Fraaije JGEM (1997) LINCS: A linear constraint solver for molecular simulations. J Comput Chem 18: 1463-1472.

13. Hoover WG (1985) Canonical dynamics: Equilibrium phase-space distributions. Phys Rev A 31: 1695-1697.

14. Nose S (1984) A unified formulation of the constant temperature molecular dynamics methods. The Journal of Chemical Physics. pp. 511-519.

15. Parrinello M, Rahman A (1981) Polymorphic transitions in single crystals: A new molecular dynamics method. J Appl Phys 52: 7182-7190.

16. Essmann U, Perera L, Berkowitz ML, Darden T, Lee H, et al. (1995) A smooth particle mesh Ewald potential. J Chem Phys 103: 8577-8592.

17. Hess B (2002) Determining the shear viscosity of model liquids from molecular dynamics simulations. The Journal of Chemical Physics 116: 209-217.
